# Directional flow in perivascular networks: mixed finite elements for reduced-dimensional models on graphs

**DOI:** 10.1007/s00285-024-02154-0

**Published:** 2024-11-07

**Authors:** Ingeborg G. Gjerde, Miroslav Kuchta, Marie E. Rognes, Barbara Wohlmuth

**Affiliations:** 1 Norwegian Geotechnical Institute, Oslo, Norway; 2 Simula Research Laboratory, Oslo, Norway; 3 Technical University of Munich, Munich, Germany

## Abstract

Flow of cerebrospinal fluid through perivascular pathways in and around the brain may play a crucial role in brain metabolite clearance. While the driving forces of such flows remain enigmatic, experiments have shown that pulsatility is central. In this work, we present a novel network model for simulating pulsatile fluid flow in perivascular networks, taking the form of a system of Stokes–Brinkman equations posed over a perivascular graph. We apply this model to study physiological questions concerning the mechanisms governing perivascular fluid flow in branching vascular networks. Notably, our findings reveal that even long wavelength arterial pulsations can induce directional flow in asymmetric, branching perivascular networks. In addition, we establish fundamental mathematical and numerical properties of these Stokes–Brinkman network models, with particular attention to increasing graph order and complexity. By introducing weighted norms, we show the well-posedness and stability of primal and dual variational formulations of these equations, and that of mixed finite element discretizations.

## Introduction

Cerebrospinal fluid (CSF) flow and transport in perivascular spaces (PVSs) is thought to play a key role for solute influx and metabolite clearance in the brain (Iliff et al. 2012; Bohr et al. 2022). PVSs are structural or functional compartments surrounding blood vessels on the brain surface (surface PVSs) and within the brain itself (parenchymal PVSs) with the vascular wall as their inner boundary. There is substantial interest in these processes due to their association with neurodegenerative diseases such as Alzheimer’s or Parkinson’s diseases (Tarasoff-Conway et al. 2015). Perivascular flow and transport have been linked to the cardiac rhythm (Iliff et al. 2013; Mestre et al. 2018; Bedussi et al. 2018; Rennels et al. 1990) and to other vasomotion patterns (van Veluw et al. 2020; Munting et al. 2023; Bojarskaite et al. 2023). However, our understanding of the drivers and directionality of these flows remains incomplete.

In recent years, computational modelling has emerged as a new approach for studying CSF flow and transport in and around the brain. Several groups have contributed to the development of network models for perivascular fluid flow, see e.g. Faghih and Sharp (2018); Rey and Sarntinoranont (2018); Tithof et al. (2019) and references therein, with an emphasis on hydraulic network models. Faghih and Sharp (2018) used network models to compute the total resistance offered by complex networks with many levels of branching. Tithof et al. (2019) derived explicit expressions for the resistance in annular and elliptic PVS cross-sections, and showed that the resistance can be computed for arbitrary cross-section shapes by a two-dimensional numerical computation. Further, Tithof et al. (2022) extended the hydraulic model to account for the coupled flow between PVS and tissue; their simulations identified low-resistance PVSs and high-resistance parenchyma as the scenario that most closely fits experimental results. A further sensitivity analysis identified the resistance of the PVS as the most sensitive parameter in simulations of parenchymal clearance (Boster et al. 2022).

These steady-state Poiseuille flow-type models typically use pressure gradients to drive fluid flow. Flow driven by cardiac-induced arterial pulsations or other vasomotion patterns call for pulsatile network models (Rey and Sarntinoranont 2018; Daversin-Catty et al. 2022). Vessel wall movement and asymmetric domains can give rise to complex flow patterns (Daversin-Catty et al. 2020; Carr et al. 2021), challenging the underlying modelling assumptions. Moreover, while vascular wall pulsations easily induce local, back-and-forth perivascular flow (Daversin-Catty et al. 2020; Kedarasetti et al. 2020), larger-scale and more complex networks of PVSs may be required to capture directional flow (Bedussi et al. 2018; Gjerde et al. 2023). All in all, hydraulic network models accounting for domain pulsatility in larger, branching networks are more complex to derive, as well as non-trivial to approximate numerically in a uniformly stable and robust manner.

Exploiting geometric structure, we here derive reduced-order computational models of pulsatile flow in PVS networks that incorporate time-dependent domain movement, axial velocity gradients, and porous structure. The shape, size and structure of the PVSs enter as computable parameters in the reduced models, while branching is handled by imposing conservative bifurcation conditions. Our modelling framework is thus applicable both for open, surface PVSs with irregular cross-sections (Min Rivas et al. 2020; Vinje et al. 2021; Eide and Ringstad 2024), and for parenchymal PVSs with more regular cross-sections, with or without a porous solid matrix (Hannocks et al. 2018). Utilizing these models, we simulate perivascular fluid flow due to arterial wall motion in synthetic and image-based vascular networks. Our in-silico experiments yield the following observations regarding PVS flow physiology.

- *Branching and heterogeneity can induce directional net flow in surface PVS networks.* Modelling surface PVSs as open annular spaces surrounding an arterial tree, with inlet and outlets freely allowing for CSF influx and efflux, we find that uniform pulsations of the vascular wall can drive directional flow. Such flow patterns are not expected for the PVS surrounding non-bifurcating vessels of constant radius (Daversin-Catty et al. 2020; Kedarasetti et al. 2020; Gjerde et al. 2023). The volume of net flow increases with the number of generations in the network and persists for arbitrary wave frequencies. These observations suggest that the network complexity may contribute to directional perivascular flow.
- *Continuous, parenchymal* perivascular networks do not form a low-resistance pathway. Modelling the parenchymal PVS as a continuous network extending continuously from arteries, to capillaries, to veins yields negligible net flow. The high resistance of capillary PVS disrupts the peristaltic interaction between the evolving pressure and resistance fields, thus inhibiting net flow generation. Thus, understanding and quantifying parenchymal PVS fluid inlets and outlets is of critical importance to understanding directional flow in this compartment.

From a mathematical point of view, the reduced-order network models take the form of a system of Stokes–Brinkman equations, which, after time-discretization, define a saddle-point problem for the perivascular cross-section fluxes and pressures. A key numerical question is then how to discretize these equations in a manner that ensures uniform well-posedness as the complexity and cardinality of the perivascular network increases. Here, we theoretically analyze and compare several variational formulations and respective mixed finite element discretizations. We show that uniform stability and robustness can be obtained with respect with appropriately-weighted norms. The final discretized models have a low computational cost, making simulation of pulsatile perivascular flow in large networks easily realizable.

This paper is structured as follows. In Sect. 2, we introduce our geometrical and physiological assumptions and present a mathematical model for pulsatile flow in branching perivascular networks. Next, we apply this model to examine the flow induced by vascular pulsations in surface and parenchymal PVS and discuss these results in their physiological context in Sect. 3. A reader primarily interested in the biological results is thus referred to Sects. 1–3. In Sect. 4, we turn to the mathematical and numerical analysis, introducing graph calculus as a framework for a unified model formulation. We formulate primal and dual semi-discrete and discrete variational formulations of the Stokes–Brinkman network models, and analyze the stability of these formulations with respect to the problem parameters and network topology.

## Modelling pulsatile fluid flow in perivascular networks

This section introduces a rigorously-derived network model describing pulsatile flow of an incompressible fluid in open or porous PVSs. This general framework encompasses both open spaces with potentially irregular cross-sections such as PVS embedded in the subarachnoid space (Min Rivas et al. 2020; Vinje et al. 2021), or porous PVSs with annular cross-sections such as those of penetrating arterioles, venules and capillaries.

### Perivascular geometry

**Fig. 1:**
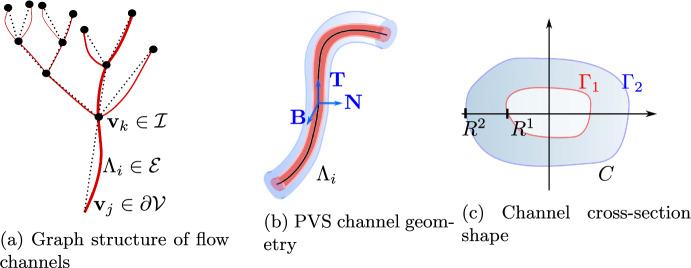
The PVS consists of a network of annular flow channels surrounding the vasculature. We organize the channels as a graph $\mathcal {G}=(\mathcal {V}, \mathcal {E})$, with internal vertices $\mathcal {I}$ and boundary vertices $\partial \mathcal {V}$. Each branch *i* of the network consists of annular generalized cylinders with centerline $\Lambda _i$. The channel cross-sections $C_i(s,t)$ are characterized by a given inner radius $R^1(s,\theta ,t)$ and outer radius $R^2(s,\theta ,t)$

We model the PVS as a network of flow channels (*branches*) surrounding the vasculature, described by a graph $\mathcal {G}$ representing the (peri)vascular centerlines and the cross-section shapes of the PVS (Fig. 1). The graph is kept fixed in time, while the cross-section shape is dynamic, allowing fluid to be pushed in, through, and out of the PVS.

#### Perivascular network structure

The network itself is represented by an oriented spatial graph $\mathcal {G} = (\mathcal {V}, \mathcal {E})$, with *m* (graph) vertices $\mathcal {V}=\{\textbf{v}_1,..., \textbf{v}_m \}$ for $\textbf{v}_j \in \mathbb {R}^3$ ($j = 1, \dots , m$) and *n* (graph) edges $\mathcal {E}= \{\Lambda _1, \Lambda _2,..., \Lambda _n \}$ connecting these vertices. We define each edge $\Lambda _i = \{\varvec{\lambda }_i(s) \} \subset \mathbb {R}^3$ ($i = 1, \dots , n$) as a $C^2$-regular curve parameterized by $\varvec{\lambda }_i: s\rightarrow \mathbb {R}^3$ for $s \in (0, \ell _i)$; letting $\vert \varvec{\lambda }_i'(s) \vert =1$ ($\vert \cdot \vert$ being the Euclidean norm) so that *s* coincides with the arc-length of the curve, and $\ell _i > 0$ denotes the edge length. Moreover, if $\Lambda _i$ connects from $\textbf{v}_j$ to $\textbf{v}_k$, $\varvec{\lambda }_i(0) = \textbf{v}_j$, and $\varvec{\lambda }_i(\ell _i) = \textbf{v}_k$. For each vertex $\textbf{v}_j \in \mathcal {V}$, we denote by $E(\textbf{v}_j)$ the set of edges connected to $\textbf{v}_j$, and by $E_\text {in}(\textbf{v}_j)$ and $E_\text {out}(\textbf{v}_j)$ the edges going into and out of vertex $\textbf{v}_j$, respectively. Note that the domains $\Lambda _i$ are open, meaning that $\mathcal {E}$ and $\mathcal {V}$ are disjoint. We denote by $\Lambda \subset \mathbb {R}^3$ the extension of the graph,

$$\begin{aligned} \Lambda = \mathcal {E}\cup \mathcal {V}, \end{aligned}$$

which is the geometric domain containing all edges and vertices. The set of vertices $\mathcal {V}$ is split into *internal vertices* $\mathcal {I}$ and *boundary vertices* $\partial \mathcal {V}$. By definition, each internal vertex is connected to two or more edges, while each boundary vertex is connected to a single edge.

#### Perivascular channels and cross-section shape

Equipped with the graph representation of the perivascular network, we now define the PVS itself by also describing the cross-sections *C* along the centerlines $\varvec{\lambda }$ via a suitable polar coordinate system (the Frenet-Serret frame (Gansca et al. 2002)). Motivated by the physiology at hand, we assume that the cross-sections of each PVS branch can be well represented by a *generalized annular* or *annular-like* domain described by inner and outer curves, representing the interface towards the blood vessel and surrounding tissue, respectively (also see Fig. 1).

More precisely, consider first a single branch with centerline $\Lambda _i$ and its Frenet–Serret frame $\textbf{T}_i, \textbf{N}_i, \textbf{B}_i$ (representing the tangent, normal and binormal directions). We then define the channel $\Omega _i = \Omega _i(t)$ by the open domain

1 $$\begin{aligned} \Omega _i= &  \{ \varvec{\lambda _i}(s) + r \cos (\theta ) \textbf{N}_i(s) + r\sin (\theta ) \textbf{B}_i(s), 0< s< \ell _i, 0 < \theta \nonumber \\\leqslant &  2\pi , R^{1}_i \leqslant r \leqslant R^2_i \}, \end{aligned}$$

where $r=r(s)$ and $\theta =\theta (s)$ are the cylindrical coordinates of the local coordinate system defined by vectors $\textbf{N}_i(s)$ and $\textbf{B}_i(s)$, and $R^1_i = R^1_i(s,\theta ,t)$ and $R^2_i = R^2_i(s, \theta ,t)$ denotes the inner and outer radii, respectively. We emphasize that these radii are allowed to vary along the centerline (with *s*) and angularly (with $\theta$) and thus should be interpreted as generalized radii and the resulting domain as a generalized annular cylinder. The cross-section $C_i = C_i(t)$ of the channel (varying along with *s*) is now given by:

2 $$\begin{aligned} C_i = \{ \varvec{\lambda }_i + r \cos (\theta ) \textbf{N}_i + r\sin (\theta ) \textbf{B}_i, 0 < \theta \leqslant 2 \pi , \, R_i^{1} \leqslant r \leqslant R_i^{2} \}. \end{aligned}$$

We let $A_i = \vert C_i \vert$ denote the cross-section area. The inner and outer lateral boundaries of $\Omega _i$ are labeled $\Gamma ^1_i$ and $\Gamma ^2_i$, respectively, and we set $\Gamma _i = \Gamma ^1_i \cup \Gamma ^2_i$.

Finally, we construct the full PVS as the union of the separate channels $\Omega = \cup _{i=1}^n \Omega _i$, with $\Omega _i$ defined by (1). We designate $\Gamma = \cup _{i=1}^n \Gamma _i$ to be its lateral boundary. Moreover, we define the perivascular cross-section area *A*, cross-section *C*, inner and outer radii $R^1$ and $R^2$, edge-wise by

$$\begin{aligned} A |_{\Lambda _i} = A_i, \quad C |_{\Lambda _i} = C_i, \quad R^1 |_{\Lambda _i} =R_i^1, \quad R^2 |_{\Lambda _i} =R_i^2. \end{aligned}$$

### Stokes–Brinkman perivascular flow equations (3D)

Consider the flow of an incompressible, viscous fluid in a saturated porous domain $\Omega \subset \mathbb {R}^3$ representing the PVS. The PVS can be open or porous, with porosity $\varphi \in (0, 1]$, with $\varphi =1$ corresponding to an open space. The porosity describes the pore space accessible to the fluid; with $\varphi = 1$ corresponding to a non-porous/open/unrestricted domain. Let $\textbf{v}^{\text {ref}}$ denote the Darcy velocity of the fluid and $p^{\text {ref}}$ a scaled fluid pressure (i.e. the pressure divided by the fluid density) solving the following Stokes–Brinkman system (Brinkman 1949) of time-dependent partial differential equations (PDEs) over $\Omega$:

3a $$\begin{aligned} \partial _t \textbf{v}^{\text {ref}}- \frac{\nu }{\varphi } \Delta \textbf{v}^{\text {ref}}+ \frac{\nu }{\kappa } \textbf{v}^{\text {ref}}+ \nabla p^{\text {ref}}&= 0, \end{aligned}$$

3b $$\begin{aligned} \nabla \cdot \textbf{v}^{\text {ref}}&= 0 . \end{aligned}$$

In (3b), $\nu$ is the kinematic fluid viscosity, and $\kappa$ the permeability of the domain (typically depending on $\varphi$). The Darcy velocity $\textbf{v}^{\text {ref}}$ is related to the fluid velocity $\textbf{v}_f^{\text {ref}}$ via $\textbf{v}^{\text {ref}}= \varphi \textbf{v}_f^{\text {ref}}$. For non-porous/open domains, we have $\varphi =1$ and $\kappa \rightarrow \infty$, in which case (3a) simplifies to the momentum equation of the time-dependent Stokes equations:

$$\begin{aligned} \partial _t \textbf{v}^{\text {ref}}- \nu \Delta \textbf{v}^{\text {ref}}+ \nabla p^{\text {ref}}= 0 \quad \text { in } \Omega \end{aligned}$$

with $\textbf{v}^{\text {ref}}=\textbf{v}_f^{\text {ref}}$ being the fluid velocity.

We augment (3b) with mixed boundary conditions as follows. First, we introduce the stress $\varvec{\sigma }^{\text {ref}}_{\textbf{n}}$ defined relative to any interface, with $\textbf{n}$ as the (outward pointing) unit normal vector, by:

4 $$\begin{aligned} \varvec{\sigma }^{\text {ref}}_{\textbf{n}}(\textbf{v}^{\text {ref}}, p^{\text {ref}}) = \left( \frac{\nu }{\kappa } \nabla \textbf{v}^{\text {ref}}- p^{\text {ref}}I\right) \cdot \textbf{n}. \end{aligned}$$

As boundary conditions at the domain inlets and outlets, we prescribe a given traction:

5 $$\begin{aligned} \varvec{\sigma }^{\text {ref}}_{\textbf{n}}(\textbf{v}^{\text {ref}},p^{\text {ref}}) = \tilde{p}^{\text {ref}} \textbf{n}\qquad \text { at } \partial \Omega \setminus \Gamma , \end{aligned}$$

which allows us, e.g., to define a given pressure drop over the length of the domain. For the sake of simplicity, we assume $\tilde{p}^{\text {ref}}$ to be constant on each considered cross-section. Next, at the inner and outer lateral boundaries, we prescribe a given fluid velocity. Let $\textbf{w}$ denote the normal wall speed defined by the rate of change of inner and outer radius in the normal direction:

6 $$\begin{aligned} \textbf{w}= {\left\{ \begin{array}{ll} \partial _t {R}^1 \textbf{n}\text { on } \Gamma _{1}, \quad & \text {(inner wall movement)}\\ \partial _t {R}^2 \textbf{n}\text { on } \Gamma _{2}. \quad & \text {(outer wall movement)} \end{array}\right. } \end{aligned}$$

In particular, we assume the inner and outer radii are known at each time point. With this in hand, we then set the fluid velocity $\textbf{v}_f^{\text {ref}}$ to match the wall velocity $\textbf{w}$ on the lateral boundaries:

7 $$\begin{aligned} \textbf{v}^{\text {ref}}= \varphi \textbf{w}\text { on } \Gamma . \end{aligned}$$

We here assume that (3b) does not degenerate to a Darcy flow equation. For the reader interested in that case, we refer to Valdes-Parada et al. (2007) for the appropriate boundary conditions.

In the simulation scenarios of Sect. 3, we consider pulsating inner wall displacements with $R^1$ varying in time ($R^1=R^1(s,\theta ,t)$) while $R^2$ is fixed in time ($R^2=R^2(s,\theta ,t=0)$). The wall movement $\textbf{w}$ is then given by experimental data; or it may be calculated using a blood flow model that accounts for arterial wall displacement (Formaggia et al. 2003).

### Stokes–Brinkman perivascular network equations

In this section, we introduce a network model for pulsatile perivascular flow; that is, a geometrically-reduced model approximation to the Stokes–Brinkman flow equations (3b) tailored to PVSs. The detailed model derivation is available in Appendix A. The main ideas are as follows.

First, we make the following assumptions on each centerline $\Lambda _i$:

8a $$\begin{aligned} p^{\text {ref}}(r, \theta ; s, t)&= p^{\text {ref}}(s, t) &  s \in \Lambda _i, t > 0, (r, \theta ) \in C_i(s, t) , \end{aligned}$$

8b $$\begin{aligned} v_s^{\text {ref}}(r, \theta ; s, t)&=\hat{v}_s^{\text {ref}}(s,t) v^{vp}(r,\theta ) &  s \in \Lambda _i, t > 0, (r, \theta ) \in C_i(s, t), \end{aligned}$$

where $v_s^{\text {ref}}$ denotes the axial component of $\textbf{v}^{\text {ref}}$. The first assumption states that the pressure is constant along each cross-section. The second states that the velocity admits a certain separation of variables, where $v^{vp}$ is the velocity profile associated with a unit pressure drop in a pipe with cross-section *C*(*s*), and $\hat{v}_s^{\text {ref}}$ gives a time-dependent scaling of this profile in the axial direction.

With these assumptions in hand, the full model equations can be integrated over the cross-section, and the derivatives moved out of the integral, yielding a one-dimensional model posed via a *cross-section flux* $q= q(s, t)$ and the *cross-section pressure* $p= p(s, t)$, defined as follows:

9 $$\begin{aligned} \begin{aligned} q= \int _{C} v_s^{\text {ref}} r \, \textrm{d} r\, \textrm{d} \theta , \quad p=\int _{C} p^{\text {ref}}\, r \, \textrm{d} r\, \textrm{d} \theta . \end{aligned} \end{aligned}$$

Here, *q* and *p* are defined by their restriction to each centerline $\Lambda _i$ and cross-section $C_i$; that is, $q=q_i$ and $p=p_i$ on $\Lambda _i$. The resulting model is a time-dependent Stokes–Brinkman equation solving for the cross-section pressure $p$ and cross-section flux *q*. For each centerline $\Lambda _i$, the model reads:

10a $$\begin{aligned} \partial _t q+ \mathcal {R}q- \nu _{\text {eff}} \partial _{ss} q+ \partial _s p&=0 &  \text { on } \Lambda _i, \end{aligned}$$

10b $$\begin{aligned} \partial _s q&= \partial _t A &  \text { on } \Lambda _i, \end{aligned}$$

where $\nu _{\text {eff}}(s,t)=\nu /(A (s, t) \varphi )$. Physically, the source term $\partial _t A$ accounts for displacement of the fluid due to wall motion. The resistance $\mathcal {R}$ is a lumped parameter varying axially and in time,

11 $$\begin{aligned} {\mathcal {R}}(s,t) = \mathcal {R}^{cs}+\mathcal {R}^{pm}, \quad \mathcal {R}^{cs} = \frac{\nu }{q^{vp}(s, t)}, \quad \mathcal {R}^{pm}=2\frac{\nu }{\kappa }, \end{aligned}$$

where $\mathcal {R}^{cs}$ denotes the resistance induced by the no-slip boundary condition and $\mathcal {R}^{pm}$ denotes the resistance induced by the PVS porosity. Here,

12 $$\begin{aligned} q^{vp} = \int _{C} v^{vp}(r,\theta ) r\, \textrm{d} r\, \textrm{d} \theta , \end{aligned}$$

is the cross-section flux associated with $v^{vp}$ in (8b); the next section will show how $v^{vp}$ can be computed for any cross-section *C*.

To formulate the model for the entire network, it remains to specify conservation or continuity conditions at the (peri)vascular junctions. At each internal vertex $\textbf{v}_j \in \mathcal {I}$, we assume the pressure *p* to be continuous, and in addition impose conservation of mass in terms of the flux *q*,

13 $$\begin{aligned} {[}\![q]\!{]} _j=0 \text { at } \textbf{v}_j \in \mathcal {I}. \end{aligned}$$

Here, ${[}\![q]\!{]} _j$ is the generalized jump of *q* at vertex $v_j$,

14 $$\begin{aligned} {[}\![q]\!{]} _j = \sum _{\Lambda _i \in E_\text {in}(\textbf{v}_j)} q_i(\textbf{v}_j)-\sum _{\Lambda _i \in E_\text {out}(\textbf{v}_j)} q_i(\textbf{v}_j). \end{aligned}$$

At each boundary vertex $\textbf{v}_j \in \partial \mathcal {V}$, we assign an axial traction condition corresponding to a cross-section average of the original boundary condition (5):

15 $$\begin{aligned} \nu _{\text {eff}}\partial _s q- p = \tilde{p}^{\text {ref}} \text { at } \textbf{v}_j. \end{aligned}$$

Comparing the three-dimensional reference model (3b) with the network equations (10), we see that the axial dissipation term $\Delta v_s^{\text {ref}}$ decomposes into two parts: $\partial _{ss} q$ and $\mathcal {R} q$. The first term, $\partial _{ss} q$, accounts for viscous dissipation of energy due to changes in the axial flow speed. In our applications, this term is generally small. In fact, it is nonzero only in specific cases of pulsatile flow. The second term, $\mathcal {R} q$, accounts for (i) energy dissipation due to the no-slip boundary condition on the inner and outer walls and (ii) resistance due to the pore network. In our applications, this term is typically large. The contribution of the no-slip condition to the network resistance gives rise to the following remark.

#### Remark 1

(Both Stokes flow and Stokes–Brinkman flow yield Stokes–Brinkman type network models) Consider the network resistance $\mathcal {R}$ defined by (11) as the sum of two contributions: the resistance inversely associated with the characteristic cross-section velocity profile $v^{vp}$ and the resistance due to the pore network. For non-porous channels, $\kappa \rightarrow \infty$; thus the latter contribution vanishes. The first term remains, meaning that the network model corresponding to Stokes flow still has a non-negative resistance $\mathcal {R}$. This resistance stems from the no-slip boundary condition on each cross-section, and depends on the shape and size of these through $v^{vp}$.

### Determination of network resistance

Consider the flow driven by a constant pressure drop through a domain with constant cross-section *C*. Inserting the separation of variables $v_s^{\text {ref}}=\hat{v}_s^{\text {ref}}(s,t) v^{vp}(r,\theta )$ into the Stokes–Brinkman equations (3b), we find that the velocity profile $v^{vp}$ associated with a cross-section *C* solves

16a $$\begin{aligned} -\frac{1}{\varphi } \Delta v^{vp}+\frac{1}{\kappa } v^{vp}&= -1 \quad &  \text { on } C, \end{aligned}$$

16b $$\begin{aligned} v^{vp}&= 0 \quad &  \text { on } \partial C. \end{aligned}$$

After solving this either analytically or numerically, one can compute the velocity profile cross-section flux $q^{vp}$ and hence the resistance $\mathcal {R}$ (11).

The resistance thus depends both on the shape and size of *C*. Their influence can be separated as follows. Let $\tilde{C}$ denote the non-dimensionalized cross-section, i.e. *C* scaled so that it has unit inner radius. Letting $\tilde{\mathcal {R}}$ denote the associated resistance, one then has  (Tithof et al. 2019)

17 $$\begin{aligned} \mathcal {R} = \tilde{\mathcal {R}}/ (R^1)^4, \end{aligned}$$

where the numerator $\tilde{\mathcal {R}}$ only depends on the shape of the domain *C*. In our computations, we typically assume the shape of *C* is fixed in time, meaning that the time-dependency of $\mathcal {R}$ enters through the denominator $(R^1)^4$.

#### Remark 2

(Perivascular porosity) As an alternative to being fully fluid-filled spaces, the PVSs and the extracellular space may be considered as porous media composed of extracellular matrix with the proteins collagen and laminin as major components (Hannocks et al. 2018). Applying the Kozeny-Carman equation, which expresses the permeability $\kappa$ in terms of porosity $\varphi$ and grain size *d*, we have

18 $$\begin{aligned} \kappa \sim \frac{(1-\varphi )^2}{\varphi ^3 d^2} \Rightarrow \mathcal {R}^{pm} \sim \frac{\nu \varphi ^3 d^2}{(1-\varphi ^2)}. \end{aligned}$$

Thus, the resistance occurring from the extracellular matrix is likely to be negligible due to the small diameters *d* of collagen fibrils. Recent studies (Mestre et al. 2022, Figure 2d) indicate that larger porous regions may also be present inside the PVS. These may have a substantial impact on the porous resistance $\mathcal {R}^{pm}$; however, due to the sparse experimental evidence detailing their structure, we leave this to future work.

## Perivascular flow simulations

In this section, we will use the network model (10) to simulate flow in physiological perivascular networks. To begin, we solve (16b) on the cross-sections of idealized and image-based pial PVSs, from which we determine the corresponding resistance. We find that the resistance is an order of magnitude lower for image-based cross-sections, compared to the idealized domains considered by e.g. Tithof et al. (2019).

Next, we show how the network model (10) can be used to generate proof-of-concept simulations of directional perivascular flow. We first address the question of whether (infinitely) long wavelength pulsations of the vascular wall can induce directional net flow in non-trivial perivascular networks (Sect. 3.2). Intriguingly, we find that this is possible in idealized branching networks with open inlets and outlets. Next, we repeat this experiment when the network extends continuously from arterial to venous side. This configuration produces purely oscillatory perivascular flow as the capillary resistance is increased. This indicates that sufficient connection routes between the PVSs and the surrounding tissue is a critical factor for arterial pumping of perivascular fluid.

For the computational experiments, we solve (10) using a first-order (implicit Euler) discretization of the time-derivative and a finite element discretization of the analogous primal formulation. The mesh refinement, number of time steps, and number of cycles were increased until the reported numbers were accurate to the first digit. For a detailed exposition of the numerical method, we refer the reader to Sect. 4.

**Fig. 2:**
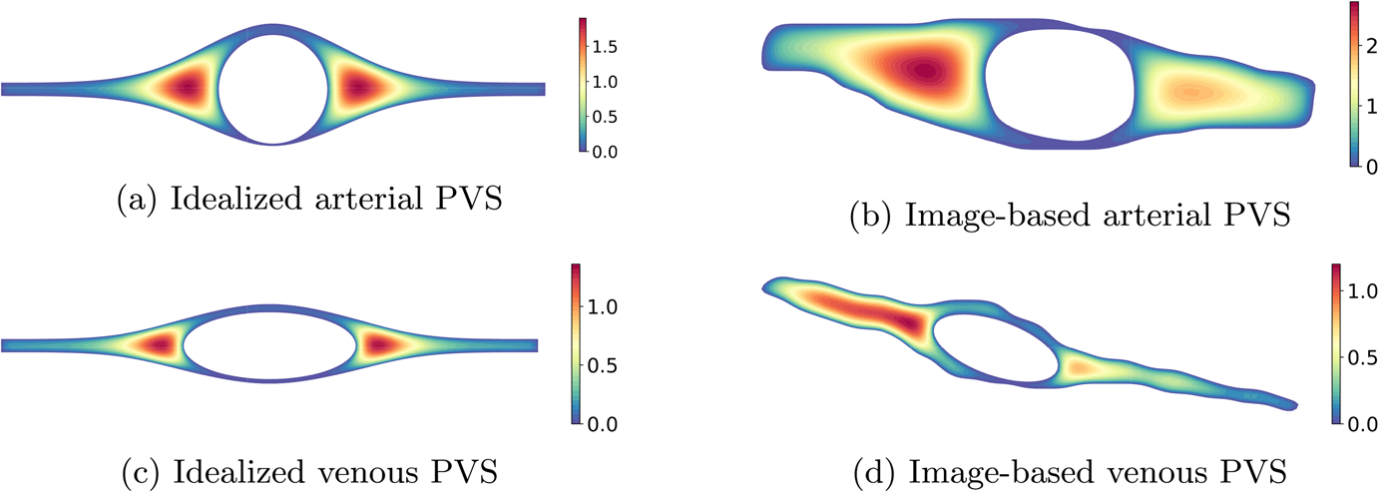
Velocity profile $v^{vp}$ associated with idealized and image-based pial PVS cross-section shapes. The top and bottom rows show shapes associated with arterial and venous PVSs, respectively. We see that the asymmetry of the image-based pial artery PVS yields an increase in the velocity profile magnitude. We therefore expect a considerably lower resistance offered by this domain

**Table 1:** The resistance parameter $\mathcal {R}$ computed for the domains shown in Fig. 2

Domain	$\mathcal {R}_{R^1=1\text {mm}^2}$	$\mathcal {R}_{A=100\text {mm}^2}$
Idealized arterial PVS	3.7e$-$03	1.2e$-$05
Image-based arterial PVS	3.5e$-$04	7.3e$-$06
Idealized venous PVS	2.0e$-$03	1.9e$-$05
Image-based venous PVS	3.5e$-$04	1.8e$-$05

The resistance parameter was computed using a reference inner radius $R^1=1$mm (middle column) and a reference area $A=100$mm$^2$ (right column). We observe that the image-based pial artery PVS yields substantially lower resistance than its idealized counterpart, even when the cross-section areas for each domain are normalized

### Resistance computation for image-based cross-sections

In Fig. 2, we show the velocity profile $v^{vp}$ computed on idealized and image-based cross-sections of pial arteries and veins, using in-vivo human image data as in (Vinje et al. 2021; Bedussi et al. 2018). In both cases, we assume that the domain is open, i.e., that $\varphi =1$. Table 1 shows the resistance parameters associated with each cross-section. The middle column gives the resistance values when the inner radius is scaled so that $R_1=1$mm. Interestingly, the image-based periarterial resistance is up to an order of magnitude lower than the resistance computed using the idealized geometries. This observation can be attributed to the effects of cross-section asymmetry, as highlighted by Tithof et al. (2019). However, resistance also decreases with cross-section area. To isolate the effect of asymmetry, we therefore show in the right-most column the resistance for cross-sections with their area normalized to 100 mm$^2$. We still observe close to 50$\%$ lower resistance.

### Long-wavelength pulsations induce directional flow in idealized perivascular networks with efflux routes

Consider a synthetic network of bifurcating blood vessels and surrounding PVSs represented by a graph $\mathcal {G}$. We assume that the network includes $N_\textrm{gen}$ generations and at baseline obeys Murray’s law; i.e., that the blood vessel radii at each junction satisfy the relation

19 $$\begin{aligned} (R^1_p)^3 = (R^1_{d_1})^3+(R^1_{d_2})^3, \end{aligned}$$

where $R^1_{p}$ and $R^1_{d_1}, R^1_{d_2}$ denote the baseline inner radii of the parent and two daughter vessels, respectively. To quantify the symmetry of the network, we introduce the branching inner radius symmetry $\gamma =R^1_{d_1}/R^1_{d_2}$. Each vessel $\Lambda _i$ is scaled such that $\ell _i=10 R^1_{i}$. For the sake of simplicity^1^, we assume that the PVS cross-sections are annular, with inner radius $R^1_i$ and outer radius $R^2_i=3R^1_i$ at baseline. Moreover, we model the PVS as non-porous ($\varphi =1$) and filled with CSF with viscosity $\nu = 1 \cdot 10^{-6}$m$^2$/s as of water. We set the root vessel radius $R^1_0 = 1$mm.

We model vascular contractions and expansions by prescribing the motion of the inner vascular wall, leaving the outer PVS boundary fixed. To isolate the effect of PVS network structure, we consider vascular pulsations in the form of uniform waves; that is, simultaneous expansions (or contractions) of the inner wall segments by

20 $$\begin{aligned} R^1(s,t) = \left( 1 + \epsilon \sin \left( \frac{t}{T_{\text {cycle}}}\right) \right) R^1(s,0) \end{aligned}$$

with amplitude $\epsilon = 0.1$ and frequency $T_{\text {cycle}}^{-1}=1$ Hz, the latter corresponding to cardiac-induced arterial pulsations (Mestre et al. 2018). These changes in the inner radius will push CSF out of (or into) the PVSs. We here allow for CSF to flow freely into the tissue via the PVS inlets and outlets by setting the fluid pressure at a reference pressure ($\tilde{p}^{\text {ref}}=0$) at all boundary vertices. Hence, we tacitly assume that CSF flow from PVS into tissue does not alter tissue pressure. Additionally, recall that changes to the inner radius will change the size of the cross-section, and hence alter the resistance field as per (11). We initialize the system at rest, $q(0)=0$.

We are interested in quantifying the net flow within the PVS network. The directional net flow $Q(s; t_1, t_2)$ through a point $\varvec{\lambda }(s)$ between the times $t_1$ and $t_2$, and its cycle-average net flow rate $\langle Q(s) \rangle$ are defined by

21 $$\begin{aligned} Q(s; t_2, t_1) = \int _{t_1}^{t_2} q (s, t) \, \textrm{d}t, \quad \langle Q(s) \rangle = \frac{1}{T_\text {cycle}} \int _{t'}^{t'+T_\text {cycle}} q(s, t) \, \textrm{d}t, \end{aligned}$$

where $T_\text {cycle}$ denotes pulsation period (time for one cycle) and $t' > 0$ is arbitrary. Naturally, the volume of fluid being displaced depends on the amplitude $\epsilon$ and the length of the vessel $\ell _i$. To measure the directionality of the displaced flow, we split *q* into oscillatory and directional parts,

22 $$\begin{aligned} q = q_{\text {osc}} +q_{\text {dir}}, \end{aligned}$$

where $q_{\text {osc}}$ is defined so that its associated net flow $\langle Q_{\text {osc}} \rangle = 0$. Next, we define the directionality ratio

23 $$\begin{aligned} \eta = \frac{\langle Q(s)\rangle }{\text {max}(q_{\text {osc}})}, \end{aligned}$$

where $\text {max}(q_{\text {osc}})$ denotes the oscillatory amplitude.

**Fig. 3:**
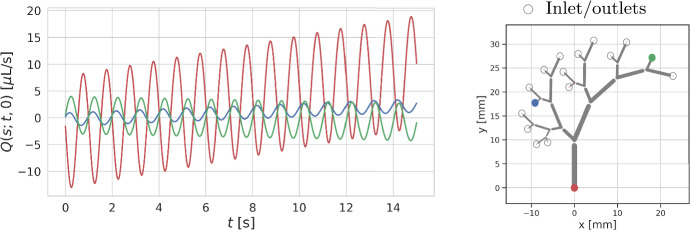
Net flow *Q*(*s*; *t*, 0) (left) over time due to uniform wave pulsations in a five-generational arterial tree (right). The tree has open inlets/outlets, and net flow is tracked through the inlet note (red) and two outlets (blue, green). Arterial pulsation can be seen to drive both oscillatory and directional flow (Color figure online)

Interestingly, these uniform waves induce oscillatory and directional flow in the Murray networks with more than one generation. Figure 3 illustrates this phenomenon in an arterial tree consisting of five generations with $\gamma = 1$. In this visual representation, we have tracked the net flow at the root node and two leaf nodes. As shown, the flow exhibits both oscillatory and directional characteristics. CSF enters via the root node and exits through leaf nodes; with one exception: the node marked in green also sees an influx of CSF.

The directional net flow depends on the perivascular network configuration (Table 2). For a one-generation network ($N_\textrm{gen} = 1$, a single vessel), the flow at the inlet remains entirely pulsatile with zero average net flow. However, the net directional flow increases with the number of network generations. Mostly, but not always, networks with larger aspect ratios ($\gamma \ll 1$) admit less net flow. We remark that as $\gamma$ is decreased, the network increasingly resembles a single vessel (with a constant inner radius), in which case uniform waves do not induce net flow (Gjerde et al. 2023). Similar experiments in networks with homogeneous radii (i.e. where all vessels are assigned the same initial radius rather than by Murray’s law) yield negligible net flow.

**Table 2:**
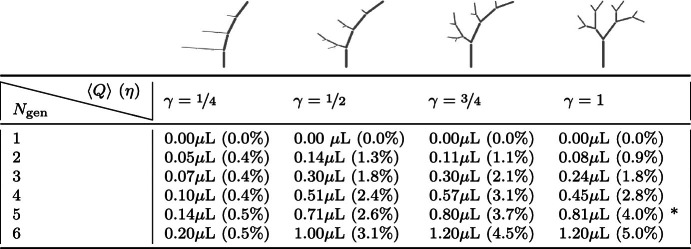
Average net flow and directionality $\eta$ (in parenthesis) at the inlet node induced by uniform inner wall waves in perivascular trees

Simulation setup is as in Fig. 3. The branching symmetry parameter $\gamma$ strongly affects the amount of net flow. The entry marked with an asterisk corresponds to the simulation visualized in Fig. 3

**Table 3:**
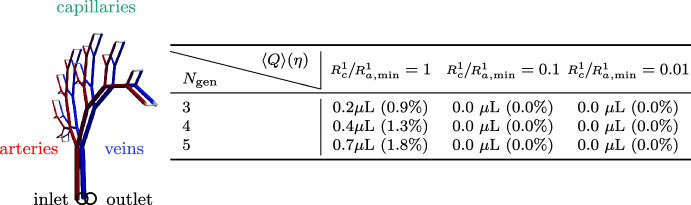
Uniform arterial contraction/expansion waves introduce negligible net flow in a connected arterial-capillary-venous PVS

The PVS is modelled as an annular space surrounding an idealized vascular network, with one arterial root node and one venous root node. As the capillary inner radius $R_c^1$ is reduced relative to the minimum arterial radius $R_{a, \text {min}}^1$, the capillary resistance increases and net flow is disrupted. This indicates that CSF influx and efflux routes are necessary to produce net flow

### Directional PVS flow due to arterial pumping is dependent on sufficient efflux routes

**Fig. 4:**
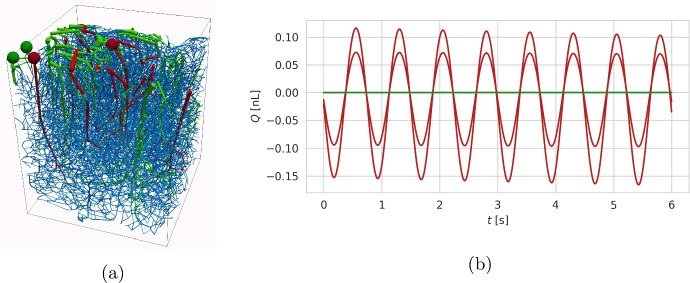
Uniform arterial pulsations create purely pulsatile flow in an image-based perivascular network extending continuously from arteries, to capillaries, to veins. The vessels are contained in a 1 mm$^3$ cube of cortical tissue; arteries are marked in red, veins in blue, and capillaries in green. Cross-section fluxes were recorded at two arterial inlets and two venous outlets, marked using red and green spheres, respectively. The flow at arterial inlets was found to be purely pulsatile. Negligible flow was recorded at venous outlets, indicating that, with this configuration, the flow induced by arterial perivascular pumping is limited to arterial vessels (Color figure online)

We now turn to consider an image-based network extracted from a 1 mm$^3$ cube of cortical tissue (Goirand et al. 2021; Blinder et al. 2013) (Fig. 4a). The network is described by the spatial locations and radius of $\sim$15,000 vessels/edges and includes 918 arteries, 216 veins, and 12559 capillaries. The PVS is modelled as a continuous space extending from arteries to veins. Arterial vessels are assigned uniform pulsations (20) with amplitude $\epsilon = 0.1$ and frequency $T_{\text {cycle}}^{-1}=1$ Hz. Capillaries and veins are assumed to have fixed radii in time.

For this configuration, the simulated flow is purely pulsatile measured at arterial inlets (red curves in Fig. 4b). This is in contrast to the results in the previous section (cf. Fig. 3), where net flow was found to occur in a network with open inlets and outlets. We hypothesize that the minuscule cross-sections of the capillary PVS connecting the arterial and venous sides play an important role for these observations. These give rise to a high resistance in the capillary part of the network. Thus, while this network is endowed with multiple inlets and outlets, the capillary PVS effectively act as a no-flow zone, thus eliminating the route for net fluid movement. Indeed, negligible net flow is observed at the venous outlets (green curves in Fig. 4b).

To better understand these observations, we repeat the simulations but in an idealized PVS network extending continuously from arteries to capillaries to veins. The arterial and venous sides are modelled as identical vascular trees with $N_\text {gen}$ generations, and connected via edges acting as capillaries (Fig.  3). To assess the impact of capillary resistance on net flow, we adjust the inner radius $R_c^1$ of the capillary vessels to be a given fraction of the minimum arterial inner radius $R_{a, \text {min}}^1$. Table 3 reports the net flow and directionality ratio $\eta$ induced by arterial pulsations with this configuration. Indeed, the directional flow component quickly vanishes as the capillary radii shrinks, increasing the resistance of the capillary vessels.

### Discussion

The observation that spatially synchronous pulsations of the blood vessel wall (uniform waves) at the frequency of cardiac pulsations (1Hz) can induce directional net flow of CSF in the PVSs is notable. Experimental observations of rapid solute transport along brain surface arteries in lockstep with the movements of the arterial walls have pointed at the presence of such perivascular flow (Mestre et al. 2018; Bedussi et al. 2018). However, these findings have been hard to reconcile with modelling based on computational fluid dynamics. Many theoretical and computational studies have found that the long wavelength of arterial wall pulsations ($\sim$100 mm) compared to the shorter typical vessel length ($\leqslant$ 1 mm) does not admit net flow by perivascular pumping (Asgari et al. 2016; Rey and Sarntinoranont 2018; Kedarasetti et al. 2020; Martinac and Bilston 2019; Daversin-Catty et al. 2020). However, if the wave length and vessel length are of comparable size, then the notion that peristaltic pumping can induce non-negligible net flow is also supported by theoretical considerations (Wang and Olbricht 2011; Thomas 2019; Gjerde et al. 2023). Thus, vascular dynamics at shorter wavelengths (and higher amplitudes) such as e.g. stimulus-evoked or spontaneous vasomotion (van Veluw et al. 2020; Munting et al. 2023) and their modulations during sleep (Bojarskaite et al. 2023) can also support net flow (Gjerde et al. 2023). Most of these theoretical or computational studies have considered single vessel segments. Our findings indicate that the network architecture plays a significant role. This concept is in agreement with our previous observations (Gjerde et al. 2023) that net flow induced by traveling vascular waves may be amplified or diminished by nonlinear network interactions.

Another key observation is that the connection between the PVS and tissue is vital to admit directional net flow. In the PVS network configurations where net flow is observed, the network inlets and outlets are open and thus allow for CSF flux into and out of the network, with negligible resistance. Conversely, when the PVS was modelled as a network continuously extending from the arterial to venous side with only a few influx and efflux routes, a collapse in net flow was observed.

In terms of modelling limitations, we consider only motion of the inner perivascular wall, ignoring the elasticity of the surrounding tissue. All simulations assume the cross-section to be an annular circle, and we do not model pressure interactions between the PVS network flux and the surrounding tissue. Moreover, we only consider CSF influx and efflux via root and leaf nodes.

Further modelling would be needed to incorporate image-based cross-sections such as those in Fig. 2, in order to understand how variations in the inner radius affect the cross-section shape. Alternatively, experimental evidence can provide precise information about the cross-section shape over time Bojarskaite et al. (2023), allowing the precise determination of the cross-section area and resistance over time.

With respect to CSF influx and outflux, the outer layer of the PVS is covered by a mosaic of astrocytic endfeet (Mathiisen et al. 2010) with inter-endfeet gaps. The endfeet or their gaps may provide an alternative route for the exchange of fluid between the PVS and the surrounding tissue. However, the parenchyma can be expected to offer significant resistance to CSF inflow (Holter et al. 2017). Further simulations are therefore necessary to assess the tractability of net PVS flow in the parenchyma. To this end, one could couple the network equation presented herein to a coupled 1d-3d flow model (D’Angelo and Quarteroni 2008), using the permeability estimates of Koch et al. (2023). Recent work (Gan et al. 2023; Bork et al. 2023) has postulated that these endfeet can act as valves, which could act as an additional mechanism driving net PVS flow. This could also be assessed with the use of a coupled 1d-3d model.

## Uniform well-posedness and approximation of Stokes–Brinkman network models

In this section, we focus on mathematical and numerical properties of the Stokes–Brinkman perivascular network equations (10). To facilitate the analysis, we introduce a graph calculus-based formulation of our network model, and we therefore first define some general concepts from graph calculus (Friedman and Tillich 2004) in Sect. 4.1, before presenting the abstract model formulation in Sect. 4.2. The well-posedness and stability of primal and dual formulations of this model are studied in Sect. 4.3 and 4.4. Importantly, we show that the formulations are uniformly stable with respect to the network topology in terms of the number of bifurcations. In Sect. 4.5, we compare and evaluate numerical properties of the primal and dual discretizations. Both methods converge with respect to the meshsize. The discretizations were implemented in FEniCS (Alnæs et al. 2015), using graphnics (Gjerde 2022) to construct the jump conditions and FEniCS_ii (Kuchta 2021) to assemble the resulting block matrices.

### Graph calculus and graph finite elements

Consider an oriented spatial graph $\mathcal {G} = (\mathcal {V}, \mathcal {E})$ with vertices $\mathcal {V}=\{\textbf{v}_1,..., \textbf{v}_m \}$ for $\textbf{v}_j \in \mathbb {R}^3$ ($j = 1, \dots , m$) and edges $\mathcal {E}= \{\Lambda _1, \Lambda _2,..., \Lambda _n \}$ parametrized by *s*. Let $C^{k}(\mathcal {E})$ denote the space of functions that are *k*-times continuous on each curve $\Lambda _i$. Further, let $L^2(\mathcal {V})$ denote the set of functions that are finite on each $\textbf{v}_j \in \mathcal {V}$.

#### Graph gradient and divergence

We define the *graph gradient* ${{\,\mathrm{\nabla _{\mathcal {G}}}\,}}: C^{k}(\mathcal {E}) \rightarrow C^{k-1}(\mathcal {E})$ by

$$\begin{aligned} {{\,\mathrm{\nabla _{\mathcal {G}}}\,}}p = \partial _s p \text { on } \mathcal {E}, \end{aligned}$$

and a *graph divergence* ${{\,\mathrm{\nabla _{\mathcal {G}} \cdot }\,}}: C^k(\mathcal {E}) \rightarrow C^{k-1}(\mathcal {E}) \times L^2(\mathcal {V})$ by

$$\begin{aligned} \nabla _{\mathcal {G}} \cdot q = \left\{ \begin{array}{l} \partial _s q \text { on } \mathcal {E}, \\ {[}\![q]\!{]} _j \text { on } \textbf{v}_j \in \mathcal {V}, \end{array}\right. \end{aligned}$$

where ${[}\![q]\!{]} _j$ is the jump of *q* defined in (14). We also define the *edge Laplacian* $\Delta _{\mathcal {E}}: C^{k}(\mathcal {E}) \rightarrow C^{k-2}(\mathcal {E})$ by

$$\begin{aligned} \Delta _{\mathcal {E}} p = \partial _{ss} p \text { on } \mathcal {E}. \end{aligned}$$

Formally, the gradient and edge Laplacian map functions from $\mathcal {E}$ to $\mathcal {E}$. The divergence maps functions from $\mathcal {E}$ to $\mathcal {G}$, where $\mathcal {G}$ consists of vertices and edges. These operators reflect the mixed-dimensional structure of the network (consisting of one-dimensional edges connected by zero-dimensional vertices), and can be seen as a special case of the operators introduced by Boon et al. (2021).

#### Sobolev spaces on graphs

We can use these differential operators to define inner products and Sobolev spaces on the graph. Recall that $\Lambda$ denotes the extended graph, i.e.,

$$\begin{aligned} \Lambda = \mathcal {V}\cup \mathcal {E}= \cup _{i=1}^n \, \text {closure}( \Lambda _i). \end{aligned}$$

Given a measurable function *u* defined over $\Lambda$, let $u_i$ denote the restriction of *u* to $\Lambda _i$. We define the inner product

$$\begin{aligned} (u,v)_\Lambda = \sum _{i=1}^n (u_i, v_i)_{\Lambda _i} =\sum _{i=1}^n \int _{\Lambda _i} u_i v_i \, \textrm{d} s, \end{aligned}$$

which gives rise to the *standard* $L^2$-space

$$\begin{aligned} L^2(\Lambda )=L^2(\mathcal {E}) = \left\{ u \text { meas.}: \int _\Lambda u^2\, \textrm{d} s< \infty \right\} . \end{aligned}$$

We note that we can identify $L^2(\Lambda )$ with $L^2(\mathcal {E})$ as they belong to the same equivalence class.

Introducing a graph measure allows us to take into account the fact that edges and vertices have different dimensions. The graph measure $\, \textrm{d}\mathcal {G}$ (Friedman and Tillich 2004) is given by

$$\begin{aligned} \int _{\mathcal {G}} u \, \textrm{d}\mathcal {G}= \int _{\mathcal {E}} u \, \textrm{d}\mathcal {E}+ \int _{\mathcal {V}} u \, \textrm{d}\mathcal {V}, \end{aligned}$$

with edge and vertex measures

$$\begin{aligned} \int _{\mathcal {E}} u \, \textrm{d}\mathcal {E}= \sum _{i=1}^n \int _{\Lambda _i} u_i \, \textrm{d} s, \quad \int _{\mathcal {V}} u \, \textrm{d}\mathcal {V}= \sum _{j=1}^m u(\textbf{v}_j). \end{aligned}$$

The graph measure thus naturally induces a graph inner product

$$\begin{aligned} (u,v)_\mathcal {G} = \sum _{i=1}^n (u,v)_{\Lambda _i} + \sum _{j=1}^m u(\textbf{v}_j) v(\textbf{v}_j), \end{aligned}$$

and the corresponding $L^2$ space

$$\begin{aligned} L^2(\mathcal {G}) = \left\{ u \text { meas. }: \sum _{i=1}^n \Vert u \Vert _{L^2(\Lambda _i)} + \sum _{j=1}^m \vert u(\textbf{v}_j)\vert ^2 <\infty \right\} . \end{aligned}$$

We will also use the notation $u = (u_\mathcal {E}, u_\mathcal {V}) \in L^2(\mathcal {G})$ to separate the edge and vertex components of *u*.

We now construct different types of Sobolev spaces on $\mathcal {G}$. We use $H^1(\mathcal {E})$ and $H^2(\mathcal {E})$ to denote the broken Sobolev spaces

$$\begin{aligned} H^1(\mathcal {E})&= \{u \in L^2(\mathcal {E}): \partial _{s} u \in L^2(\mathcal {E}) \}, \\ H^2(\mathcal {E})&= \{u \in L^2(\mathcal {E}): \partial _{s} u \in L^2(\mathcal {E}), \partial _{ss} u \in L^2(\mathcal {E}) \}, \end{aligned}$$

and $H^1(\Lambda )$ is defined as:

$$\begin{aligned} H^1(\Lambda ) = \{ u \in L^2(\Lambda ): {{\,\mathrm{\nabla _{\mathcal {G}}}\,}}u \in L^2(\Lambda )\}. \end{aligned}$$

The latter space is known from e.g. (Arioli and Benzi 2018), and has the norm

$$\begin{aligned} \Vert u \Vert _{H^1(\Lambda )}^2 = \Vert u \Vert _{L^2(\Lambda )}^2 + \Vert {{\,\mathrm{\nabla _{\mathcal {G}}}\,}}u \Vert _{L^2(\Lambda )}^2. \end{aligned}$$

We use the notation $H_0^1(\Lambda )$ to denote $H^1$-functions with zero trace on $\partial V$. While $L^2(\Lambda )$ is equivalent to $L^2(\mathcal {E})$, we note that $H^1(\mathcal {E})$ and $H^1(\Lambda )$ are not equivalent. Indeed, recalling from standard Sobolev theory that $H^1(\Lambda )\subset C^0(\Lambda )$, we find $H^1(\Lambda ) \subset H^1(\mathcal {E})$, as $H^1(\mathcal {E})$ functions can be discontinuous across vertices.

Next, let $H(\textrm{div};\mathcal {G})$ denote the space

$$\begin{aligned} H(\textrm{div}; \mathcal {G}) = \{ q \in L^2(\mathcal {\mathcal {E}}): {{\,\mathrm{\nabla _{\mathcal {G}} \cdot }\,}}q \in L^2(\mathcal {G})\}, \end{aligned}$$

with the norm

24 $$\begin{aligned} \Vert q \Vert _{H(\textrm{div};\mathcal {G})}^2= &  \Vert q \Vert _{L^2(\mathcal {E})}^2 + \Vert {{\,\mathrm{\nabla _{\mathcal {G}} \cdot }\,}}q \Vert _{L^2(\mathcal {G})}^2 \nonumber \\= &  \sum _{i=1}^n \Vert q \Vert _{L^2(\Lambda _i)}^2 + \sum _{i=1}^n \Vert \partial _s q \Vert _{L^2(\Lambda _i)}^2+ \sum _{j=1}^m \vert {[}\![q]\!{]} _j \vert ^2. \end{aligned}$$

**Fig. 5:**
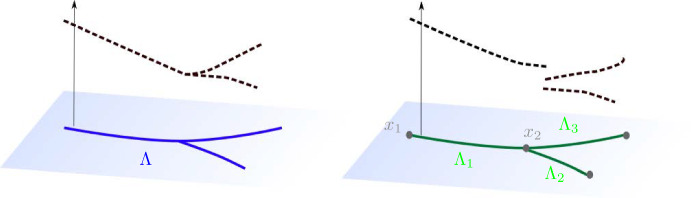
Examples of functions that are in $H^1(\Lambda )$ (left) and $H(\textrm{div}; \mathcal {G})$ (right)

Figure 5 shows examples of functions in $H^1(\Lambda )$ versus $H(\textrm{div};\mathcal {G})$. The main difference between these spaces is that $u \in H(\textrm{div}; \mathcal {G})$ can be discontinuous at the vertices. We note that $H^1(\mathcal {E})$ and $H(\textrm{div};\mathcal {G})$ are equivalent, as $H^1(\mathcal {E})$-functions have bounded values at $\partial \Lambda _i$ (and hence bounded jumps across $\mathcal {V}$). However, we keep the $H(\textrm{div};\mathcal {G})$-notation to emphasize the connection to standard methods for dual mixed formulations. Moreover, we will see that an appropriately weighted $H(\textrm{div};\mathcal {G})$-norm is required for uniform stability.

Having $H^1(\Lambda )$ and $H(\textrm{div};\mathcal {G})$ defined, it is easy to see that the following integration by parts formula holds.

##### Lemma 4.1

(Integration by parts) For $p\in H^1_0(\Lambda )$ and $q\in H(\textrm{div};\mathcal {G})$ there holds that

$$\begin{aligned} \int _\mathcal {G} ({{\,\mathrm{\nabla _{\mathcal {G}} \cdot }\,}}q) \, p \, \textrm{d}\mathcal {G}= -\int _{\mathcal {E}} q \, ({{\,\mathrm{\nabla _{\mathcal {G}}}\,}}p) \, \textrm{d}\mathcal {E}. \end{aligned}$$

##### Proof

A direct calculation shows that

$$\begin{aligned} \begin{aligned} - \int _{\mathcal {E}} q \, ({{\,\mathrm{\nabla _{\mathcal {G}}}\,}}p) \, \textrm{d}\mathcal {E}&= - \sum _{i=1}^n \int _{\Lambda _i} q_i \, \partial _s p_i \, \textrm{d} s= \sum _{i=1}^n \int _{\Lambda _i} \partial _s q_i \, p_i \, \textrm{d} s+ \sum _{j=1}^m {[}\![pq]\!{]} _j \\&= \sum _{i=1}^n \int _{\Lambda _i} \partial _s q\, p \, \textrm{d} s+ \sum _{j=1}^m p(v_j) {[}\![q]\!{]} _j = \int _\mathcal {G} ({{\,\mathrm{\nabla _{\mathcal {G}} \cdot }\,}}q) p \, \textrm{d}\mathcal {G}, \end{aligned} \end{aligned}$$

where we used that *p* is continuous over the graph. $\square$

#### Finite element spaces on graphs

We now introduce finite element meshes and finite element spaces defined relative to the graph. Let $\Lambda ^h$ be a finite element mesh of the centerline $\Lambda$, composed of mesh segments $\Lambda _i^h$, one for each centerline $\Lambda _i$. Each mesh segment $\Lambda _i^h$ is a mesh consisting of intervals embedded in $\mathbb {R}^3$. Relative to $\Lambda _i^h$, we define $CG_{k}(\Lambda _i^h)$ to be the space of continuous piecewise polynomials of degree *k*,i.e.

$$\begin{aligned} CG_{k}(\Lambda _i^h) = \lbrace v^h \in C^0(\Lambda _i), \, v^h \vert _T \in P_k(T) \text { for } T \in \Lambda _i^h \rbrace , \end{aligned}$$

and similarly $CG_{k}(\Lambda ^h)$ to be the space of continuous piecewise polynomials of degree *k* defined relative to $\Lambda ^h$, i.e.

$$\begin{aligned} CG_{k}(\Lambda ^h) = \lbrace v^h \in C^0(\Lambda ), \, v^h \vert _T \in P_k(T) \text { for } T \in \Lambda ^h \rbrace . \end{aligned}$$

We define $DG_{k}(\Lambda _i^h)$ to be the space of discontinuous piecewise polynomials of degree *k* on $\Lambda _i^h$, i.e.

$$\begin{aligned} DG_{k}(\Lambda _i^h) = \lbrace v^h \in L^2(\Lambda _i), \, v^h \vert _T \in P_k(T) \text { for } T \in \Lambda _i^h \rbrace , \end{aligned}$$

and $DG_{k}(\Lambda ^h) = \cup _{i=1}^n DG_{k}(\Lambda _i^h)$ to be the analogous space on $\Lambda ^h$.

### A graph calculus formulation of the Stokes–Brinkman network model

With the graph calculus notation introduced in Sect. 4.1.1, the time–dependent Stokes–Brinkman model (10) can be succinctly expressed as: for $t > 0$, find $(q, p)$ defined over $\mathcal {G}$ such that

25a $$\begin{aligned} \partial _t q+ \mathcal {R} q- \nu _{\text {eff}} \Delta _\mathcal {E} q+ {{\,\mathrm{\nabla _{\mathcal {G}}}\,}}p&= 0 &  \text { on } \mathcal {E}, \end{aligned}$$

25b $$\begin{aligned} {{\,\mathrm{\nabla _{\mathcal {G}} \cdot }\,}}q&= f &  \text { on } \mathcal {G} , \end{aligned}$$

where

26 $$\begin{aligned} f = {\left\{ \begin{array}{ll} \partial _t A \text { on } \mathcal {E}, \\ 0 \text { on } \mathcal {V}. \end{array}\right. } \end{aligned}$$

Moreover, this system can be reduced to a time-dependent hydraulic network model. Since $\Delta _\mathcal {E}q = {{\,\mathrm{\nabla _{\mathcal {G}}}\,}}({{\,\mathrm{\nabla _{\mathcal {G}} \cdot }\,}}q)$ on the edges $\mathcal {E}$, (25b) gives that $\Delta _\mathcal {E} q= \nabla _\mathcal {G} f$. Thus, $(q, p)$ solving (25) also solve:

27a $$\begin{aligned} \partial _t q+ \mathcal {R} q+ \nabla _{\mathcal {G}} p&= g &  \text { on } \mathcal {E}, \end{aligned}$$

27b $$\begin{aligned} {{\,\mathrm{\nabla _{\mathcal {G}} \cdot }\,}}q&= f &  \text { on } \mathcal {G}, \end{aligned}$$

where by definition $g = \nu _{\text {eff}} {{\,\mathrm{\nabla _{\mathcal {G}}}\,}}f = \nu _{\text {eff}} \partial _s \partial _t A$.

#### Remark 3

(Relation to quantum graphs) The system (27) can be interpreted as a quantum graph with the differential operator $(q,p)\mapsto (\partial _t q + \mathcal {R}q + \partial _s p, \partial _s q)$. The bifurcation condition is equivalent to the standard Neumann-Kirchhoff conditions. In the stationary case, i.e. $\partial _t q=0$, the flux can be eliminated, yielding the simpler system

28 $$\begin{aligned} - \partial _s( \mathcal {R}^{-1} \partial _{s} p) = \tilde{f} \qquad \text { on } \Lambda _i, \end{aligned}$$

with $\tilde{f}= f - \partial _s (\nu _{\text {eff}} \mathcal {R}^{-1} \partial _s f).$ This corresponds to a quantum graph with the Laplacian $p\mapsto - \partial _s( \mathcal {R}^{-1} \partial _{s} p)$ as the differential operator (Berkolaiko and Kuchment 2013). The analysis we provide herein can be viewed as an extension of previous work on quantum graphs (Arioli and Benzi 2018) to the case where the differential operator is the primal and dual mixed Laplacian.

In the following sections, we will study the well-posedness and stability of (discretizations of) the hydraulic network model (27) and in part (25). To simplify the exposition, we will only consider the stationary case ($\partial _t q=0$) with homogeneous Dirichlet boundary conditions ($p=0$ on $\partial \mathcal {V}$). We will use the saddle point theory from  (Boffi et al. 2013), expressing the models in the general abstract mixed form: find $q \in V$, $p \in M$ such that

29 $$\begin{aligned} \begin{aligned} a(q, \psi ) + b (\psi , p)&= L(\psi ), \\ b\left( q, \phi \right)&= F(\phi ), \end{aligned} \end{aligned}$$

for all $\psi \in V$, $\phi \in M$. Here *V* and *M* are Hilbert spaces with inner products $(\cdot , \cdot )_V$ and $(\cdot , \cdot )_M$, respectively, $a: V \times V \rightarrow \mathbb {R}$ and $b: V \times M \rightarrow R$ are bilinear forms, and $L \in V^{*}$ and $F \in M^{*}$ are given functionals. We can and will study the hydraulic network formulation (27) in both primal and dual variational form, while the Stokes–Brinkman model (25) requires the dual form.

Given the discrete function spaces $V^h$ and $M^h$, with inner products $(\cdot , \cdot )_{V^h}$ and $(\cdot , \cdot )_{M^h}$, and norms $\Vert \cdot \Vert _{V^h}$ and $\Vert \cdot \Vert _{M^h}$, we will also consider discretizations of (29) i.e. the problem of finding discrete solutions $(q^h, p^h) \in V^h \times M^h$ such that (29) holds for all $\psi \in V^h$ and $\phi \in M^h$. The discrete system is then associated with a discrete inf-sup constant $\beta ^h$, defined by

30 $$\begin{aligned} \beta ^h = \inf _{0 \ne (q^h, p^h) \in W^h}\sup _{0 \ne (\psi , \phi ) \in W^h} \frac{\vert a(q^h, \psi ) + b(q^h, \phi )+b(\psi , p^h) \vert }{(\Vert q^h \Vert _{V^h}+\Vert p^h \Vert _{M^h})(\Vert \psi \Vert _{V^h}+\Vert \phi \Vert _{M^h})}, \end{aligned}$$

where $W^h = V^h \times M^h$. The discretization is said to be inf-sup stable if there exists some $\beta > 0$ such that $\beta ^h\ge \beta$ for any $h>0$. The inf-sup constant can be equivalently expressed as $\beta ^h = \vert \xi ^h_{\text {min}} \vert$ where $\xi ^h_{\text {min}}$ is the smallest in modulus eigenvalue of the following generalized eigenvalue problem: find $(q^h, p^h) \in W^h$, $\xi ^h\in \mathbb {R}$ such that

31 $$\begin{aligned} a(q^h, \psi ) + b(q^h, \phi )+b(\psi , p^h) = \xi ^h \left( (q^h, \psi )_{V^h} + (p^h, \phi )_{M^h} \right) \end{aligned}$$

for all $(\psi , \phi ) \in W^h$. We will use this eigenvalue problem to provide numerical evidence that finite element discretizations are uniformly stable with respect to both *h* and the network topology.

### Well-posedness of primal formulations of the hydraulic network models

In this section, we focus on the primal formulation and its stability properties.

#### A primal formulation of the hydraulic network model

We begin by presenting a primal mixed formulation of the stationary ($\partial _t q = 0$) hydraulic network model (27) with homogeneous Dirichlet conditions ($p=0$ on $\partial \mathcal {V}$) based on the function space pairing $L^2(\Lambda ) \times H^1_0(\Lambda )$. Multiplying (27) by test functions $\psi \in L^2(\Lambda )$ and $\phi \in H^1_0(\Lambda )$, integrating over the graph, and using the integration by parts (Lemma 4.1), give the *primal mixed variational formulation*: find $q \in L^2(\Lambda )$, $p \in H_0^1(\Lambda )$ such that

32 $$\begin{aligned} \begin{aligned} (\mathcal {R} q,\psi )_\mathcal {E} + (\nabla _\mathcal {G} p, \psi )_\mathcal {E}&= (g,\psi )_\mathcal {E}, \\ (q, \nabla _\mathcal {G} \phi )_\mathcal {E}&= (- f,\phi )_\mathcal {G}. \end{aligned} \end{aligned}$$

Note that for any $u,v \in L^2(\mathcal {E})$,

33 $$\begin{aligned} (u,v)_\mathcal {E}= \sum _{i=1}^n (u,v)_{L^2(\Lambda _i)} = (u, v)_{L^2(\Lambda )}. \end{aligned}$$

We thus observe that (32) fits the general abstract framework (29) when identifying $V = L^2(\Lambda )$ and $M = H^1_0(\Lambda )$, and

$$\begin{aligned} a(q, \psi ) = (\mathcal {R} q,\psi )_\Lambda , \quad b(\psi , p) = (\psi , \partial _s p)_\Lambda , \quad L(\psi ) = (g,\psi )_\Lambda , \quad F(\phi ) = (-f, \phi )_\Lambda . \quad \end{aligned}$$

Our first theoretical result shows that the system (32) is well-posed, with uniform stability and inf-sup constants in resistance-weighted norms.

##### Theorem 1

Let $V=L^2(\Lambda )$ and $M=H^1_0(\Lambda )$ be endowed with the weighted norms

34 $$\begin{aligned} \begin{aligned} \Vert p \Vert _{M}&= \Vert \mathcal {R}^{-1/2} \nabla _\mathcal {G} p \Vert _{L^2(\Lambda )}^2, \\ \Vert q \Vert _{V}&= \Vert \mathcal {R}^{1/2} q \Vert _{L^2(\Lambda )}. \end{aligned} \end{aligned}$$

Given $f \in L^2(\mathcal {G})$ and $g \in L^2(\Lambda )$, there then exists a unique solution $q \in V$ and $p \in M$ to the primal mixed variational formulation (32). Moreover, the coercivity and inf-sup constants are uniform with respect to the size and topology of the network.

##### Proof

The proof is by verifying the Brezzi conditions. First, note that by definition

35 $$\begin{aligned} a(q,q) = \Vert \mathcal {R}^{1/2}q \Vert _{L^2(\Lambda )}^2 = \Vert q \Vert _{V}^2, \end{aligned}$$

which yields coercivity of the form *a* independent of $\mathcal {G}$ with the coercivity constant equal to one. Due to the boundary conditions the Poincaré inequality guarantees that $\Vert \mathcal {R}^{-1/2} {{\,\mathrm{\nabla _{\mathcal {G}}}\,}}\phi \Vert _{L^2(\Lambda )}$ is a norm on *M*. Then, for any $\phi \in M$, letting $q = \mathcal {R}^{-1}{{\,\mathrm{\nabla _{\mathcal {G}}}\,}}\phi \in L^2(\Lambda )$, we find that by definition

36 $$\begin{aligned} \begin{aligned} \sup _{ q\in V} \frac{b(q,\phi )}{\Vert \phi \Vert _{M}}&\geqslant \frac{ \Vert \mathcal {R}^{-1/2} \nabla _{\mathcal {G}} \phi \Vert ^2_{L^2(\Lambda )}}{\Vert \phi \Vert _{M}} \geqslant \frac{ \Vert \mathcal {R}^{-1/2} \nabla _{\mathcal {G}} \phi \Vert ^2_{L^2(\Lambda )}}{\Vert \mathcal {R}^{-1/2} {{\,\mathrm{\nabla _{\mathcal {G}}}\,}}\phi \Vert _{L^2(\Lambda )}} = \Vert \phi \Vert _{M}. \end{aligned} \end{aligned}$$

This confirms the inf-sup condition with constant 1. $\square$

Applying standard Sobolev theory, we can show that the solution exhibits a higher regularity on each edge:

##### Theorem 2

(Higher regularity) Let $p \in M$, $q \in V$ solve (32). Then $p\in H^2(\mathcal {E})$ and $q\in H^1(\mathcal {E})$.

##### Proof

The proof is by a post-processing of the solution. On each edge $\Lambda _i$, define $\tilde{q}_i, \tilde{p}_i$ as the solutions of

37 $$\begin{aligned} \tilde{q}_i + \partial _s \tilde{p}_i&= f_\mathcal {E}\text { on } \Lambda _i, \end{aligned}$$

38 $$\begin{aligned} \partial _s \tilde{q}_i&= p \text { on } \partial \Lambda _i. \end{aligned}$$

This problem is well defined as $p \in H^1(\Lambda )$, meaning that *p* has a well-defined trace at the vertices. As $f_\mathcal {E}\in L^2(\Lambda _i)$, we further have $\tilde{p} \in H^2(\Lambda _i)$ and $\tilde{q}=H^1(\Lambda _i)$. By construction, $\tilde{p}_i=p$ and $\tilde{q}_i=q$ on each edge $\Lambda _i$, meaning that $p \in H^2(\mathcal {E})$ and $q \in H^1(\mathcal {E})$. $\square$

#### Stability of a family of primal discretizations

Next, we propose to discretize the primal formulation (32) using $CG_k$ spaces for pressure and $DG_{k-1}$ spaces for the flux defined relative to $\Lambda ^h$ for $k \ge 1$, e.g.:

39 $$\begin{aligned} M^h = CG_k(\Lambda ^{h}), \quad V^h = DG_{k-1}(\Lambda ^{h}). \end{aligned}$$

Assuming that $\mathcal {R}^{-1}$ is piecewise constant these spaces satisfy the discrete Brezzi stability conditions.

##### Remark 4

(Connection to finite volume schemes) Using the finite element spaces (39) with $k=1$, the primal mixed formulation can be interpreted as a staggered grid finite volume scheme (Greyvenstein and Laurie 1994) where the pressure and flux variables are bound to nodes and edges, respectively. The matrix representation of the discrete problem then takes the form

40 $$\begin{aligned} \begin{bmatrix} \pmb {\mathcal {R}} & \pmb {G}\\ -\pmb {D} & \pmb {0}\\ \end{bmatrix} \end{aligned}$$

where the matrix $\pmb {G}$, the discrete gradient/incidence matrix, encodes in its rows the connectivity of graph edges to nodes. Furthermore, $\pmb {D}$ is the transpose of $\pmb {G}$ and $\pmb {\mathcal {R}}$ is a diagonal matrix of resistances for each edge of the graph. The Schur complement $-\pmb {D} \pmb {\mathcal {R}}^{-1} \pmb {G}$ is in fact the graph Laplacian, cf. Remark 3.

**Fig. 6:**
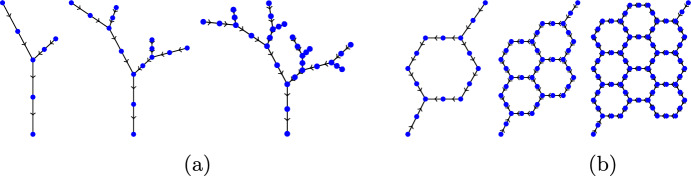
The **a** arterial *tree* and **b** *honeycomb* networks used for numerical experiments. From left to right, the networks grow by the addition of more edges. The arterial tree networks are grown by adding more generations; while the honeycomb networks grow by increasing the number of cycles

The norms (34) induce an exact Schur complement preconditioner for the discretization by (39). Since the stability constants in Theorem 1 are independent of both the graph geometry and the graph topology, we expect the condition number of (31) (i.e. $|\xi ^h_{\text {max}}|/|\xi ^h_{\text {min}}|$ with $\xi ^h_{\min }$ and $\xi ^h_{\max }$ denoting the smallest and largest in magnitude eigenvalues, respectively) to be constant for any $\mathcal {G}$ and mesh size. This theoretical expectation is confirmed by numerical experiments, see Table 4 for the case $\mathcal {R} = 1$ and the associated Fig. 6. We remark that these results would not be altered by varying the resistance.

**Table 4:**
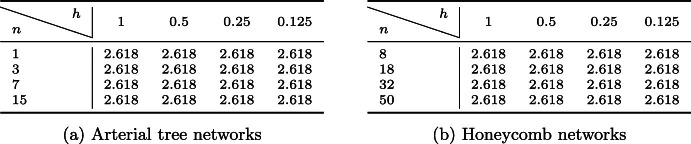
Spectral condition numbers of the generalized eigenvalue problem (31) with (referring to the notation introduced therein) *a* being the primal mixed formulation and the norms on $V^h$, $M^h$ given by (34)

Computations were performed on arterial tree and honeycomb networks (see Fig. 6), with *n* denoting the number of bifurcation vertices, and *h* denoting the mesh size

### Well-posedness of a dual mixed formulation

We now turn to introduce and analyze a dual mixed formulation of the hydraulic network model (27). The Stokes–Brinkman network model (25) can be expressed in a similar dual mixed form, as we also illustrate below but do not analyze further.

#### A dual mixed formulation of the network flow models

To construct a dual mixed variational formulations of (27), we multiply (27a) by a test function $\psi \in H(\textrm{div}; \mathcal {G})$ and (27b) by a test function $\phi \in L^2(\mathcal {G})$ and integrate over $\mathcal {G}$. Multiplication in $L^2(\mathcal {G})$ implies that we multiply edge variables by edge variables, and vertex variables by vertex variables. We then find that the hydraulic network model can be expressed in the abstract form (29) with $V = H(\textrm{div}, \mathcal {G})$ and $M = L^2(\mathcal {G})$ after defining

41a $$\begin{aligned} a(q,\psi )&= (q, \psi )_\mathcal {E}, \end{aligned}$$

41b $$\begin{aligned} b(q, \phi )&= - ({{\,\mathrm{\nabla _{\mathcal {G}} \cdot }\,}}q, \phi )_{\mathcal {G}} = - ( \partial _s q, \phi )_{\mathcal {E}} - ( {[}\![q]\!{]} , \phi )_{\mathcal {V}}, \end{aligned}$$

41c $$\begin{aligned} L(\psi )&= (g, \psi )_\mathcal {E}+ (\tilde{p}^{\text {ref}}, \psi )_{\partial \mathcal {V}}, \end{aligned}$$

41d $$\begin{aligned} F(\phi )&= -(f , \phi )_{\mathcal {G}}, \end{aligned}$$

where the second term in *b* accounts for the conservation of mass condition at the bifurcations. Further, $\tilde{p}^{\text {ref}}$ is given by the boundary conditions. We note that the Stokes–Brinkman network model (25) can be expressed in an analogous dual mixed form over $H^1(\mathcal {E}) \times L^2(\mathcal {G})$ with *b*, *L* and *F* given by (41), and *a* defined by

42 $$\begin{aligned} a(q,\psi ) = ({{\,\mathrm{\nabla _{\mathcal {G}}}\,}}\nu _{\text {eff}} q, {{\,\mathrm{\nabla _{\mathcal {G}}}\,}}\psi )_{\mathcal {E}} + (q, \psi )_{\mathcal {E}}. \end{aligned}$$

The next result shows that the dual formulation (41) is well-posed. Moreover, its stability and inf-up constants defined relative to suitably weighted norms are uniform with respect to the graph topology and cardinality.

##### Theorem 4.1

Consider the dual mixed hydraulic network problem given by (29) with (41) defined over $V=H(\textrm{div}; \mathcal {G})$ and $M=L^2(\mathcal {G})$ endowed with the weighted norms:

43a $$\begin{aligned} \Vert q \Vert _{V}^2&= \Vert q \Vert _{L^2(\mathcal {E})}^2 + \Vert \ell \partial _s q \Vert _{L^2(\mathcal {E})}^2 + \Vert \alpha ^{-1} \ell {[}\![q]\!{]} \Vert _{L^2(\mathcal {V})}^2, \end{aligned}$$

43b $$\begin{aligned} \Vert p \Vert _{M}^2&= \Vert \ell ^{-1}p_\mathcal {E} \Vert _{L^2(\mathcal {E})}^2 + \Vert \alpha \ell ^{-1}p_\mathcal {V} \Vert _{L^2(\mathcal {V})}^2, \end{aligned}$$

where $\ell = \sum _{i=1}^n \ell _i$ is the total length of the network, and $\alpha$ is defined for each vertex $\textbf{v}_j$ as the square root of an averaged edge length:

44 $$\begin{aligned} 4 \alpha _j^2 = \left( \frac{\sum _{\Lambda _i \in E(\textbf{v}_j)} \ell _i}{m} \right) , \end{aligned}$$

where *m* is the total number of vertices in the network. Given $f \in L^2(\mathcal {G})$ and $g \in L^2(\mathcal {E})$, there then exists a unique solution $(q, p) \in V \times M$. Moreover, the Brezzi coercivity and inf-sup constants are uniform with respect to the size and topology of the network.

##### Proof

It is straightforward to show that the forms *a* and *b* are uniformly continuous with respect to the weighted norms on *V* and *M*. Next, we show that *a* is uniformly coercive on the kernel $K \subset V$ defined by:

45 $$\begin{aligned} K = \{ \psi \in V: b(\psi , p)&=0 \text { for all } p\in M \}. \end{aligned}$$

Consider any $\psi \in K$, and take $p_{\psi }=(p_\mathcal {E}, p_{\mathcal {V}}) \in L^2(\mathcal {G})$ with $p_\mathcal {E}= \ell ^2 \partial _s \psi$ and $p_\mathcal {V}=0$. A calculation then shows that

46 $$\begin{aligned} b(\psi , p_\psi )=(\nabla _\mathcal {G}\cdot \psi ,p_\psi )_\mathcal {G} = \Vert \ell \partial _s \psi \Vert _{L^2(\mathcal {E})}^2 = 0 . \end{aligned}$$

Similarly, taking $p_\mathcal {E}= 0$ and $p_\mathcal {V}=l^2 \alpha ^{-2} {[}\![\psi ]\!{]}$ gives that

47 $$\begin{aligned} b(\psi , p_\psi )=(\nabla _\mathcal {G}\cdot \psi ,p_\psi )_\mathcal {G} = \Vert l \alpha ^{-1} {[}\![\psi ]\!{]} \Vert _{L^2(\mathcal {V})}^{2} = 0 . \end{aligned}$$

Thus,

48 $$\begin{aligned} a(\psi , \psi ) = \Vert \psi \Vert _{L^2(\mathcal {E})}^2 = \Vert \psi \Vert _{L^2(\mathcal {E})}^2 + \Vert \ell \partial _s \psi \Vert _{L^2(\mathcal {E})}^2 + \Vert \ell \alpha ^{-1} {[}\![\psi ]\!{]} \Vert _{L^2(\mathcal {V})}^2 = \Vert \psi \Vert _{V}^2 , \end{aligned}$$

and *a* is uniformly coercive on *K*.

It remains to show that the inf-sup condition holds; i.e., that there exists a $\beta > 0$ such that

49 $$\begin{aligned} \sup _{\psi \in V} \frac{b(\psi ,p)}{\Vert \psi \Vert _{V}} \ge \beta \Vert p \Vert _{M} \text { for all } p\in M . \end{aligned}$$

The proof is by construction of a suitable $\psi ^p \in V$ so that

50 $$\begin{aligned} \Vert \psi ^p \Vert _{V} \lesssim \Vert p \Vert _{M} \end{aligned}$$

where we use $\lesssim$ to denote $\Vert \psi ^p \Vert _{V} \le C \Vert p \Vert _{L^2(\mathcal {G})}$ for some constant $C>0$ that is independent of the domain. To this end, fix $p \in M$. By Theorem 1 and 2, there exists $\psi ^p \in H^1(\mathcal {E})$ and $\phi ^p \in H^1(\Lambda ) \cap H^2(\mathcal {E})$ solving

51 $$\begin{aligned} \begin{aligned} \psi ^p + \nabla _\mathcal {G} \phi ^p&= 0 &  \text { on } \mathcal {E}, \\ \nabla _\mathcal {G} \cdot \psi ^p&= \ell ^{-2} p &  \text { on } \mathcal {G}. \end{aligned} \end{aligned}$$

To show (50), recall that

52 $$\begin{aligned} \Vert \psi ^p \Vert _{V}^2&= \Vert \psi ^p \Vert _{L^2(\mathcal {E})}^2+ l^{2} \Vert \partial _s \psi ^p \Vert _{L^2(\mathcal {E})}^2 + l^{2} \Vert \alpha ^{-1} {[}\![\psi ^p]\!{]} \Vert _{L^2(\mathcal {V})}^2. \end{aligned}$$

To bound the first term, note that for each edge $\Lambda _i$, there exists a $C_{s, i} > 0$, such that $\Vert \phi ^p \Vert _{H^1(\Lambda _i)} \le C_{s,i} \Vert \ell ^{-2} p \Vert _{L^2(\Lambda _i)}$ for each edge $\Lambda _i$. Here, $C_{s, i}$ depend on the Poincaré constant $C_{p, i}$ of the domain $\Lambda _i$, and $C_{p, i} \sim \ell _i$ (Arnold and Rognes 2009; Kennedy et al. 2016). Thus

53 $$\begin{aligned} \Vert \psi ^p \Vert _{L^2(\mathcal {E})}^2= &  \Vert \partial _{s} \phi ^p \Vert _{L^2(\mathcal {E})}^2 \le \Vert \phi ^p \Vert _{H^1(\mathcal {E})}^2 \le \sum _{i=1}^n C_{s,i}^2 \Vert \ell ^{-2} p \Vert _{L^2(\Lambda _i)}^2 \nonumber \\\lesssim &  \sum _{i=1}^n \ell _i^2 \Vert \ell ^{-2} p \Vert _{L^2(\Lambda _i)}^2 \le \Vert \ell ^{-2} p \Vert _{L^2(\mathcal {E})}^2 \sum _{i=1}^n \ell _i^2 \le \ell ^2 \Vert \ell ^{-2} p \Vert _{L^2(\mathcal {E})}^2 \nonumber \\= &  \Vert \ell ^{-1} p \Vert _{L^2(\mathcal {E})}^2. \end{aligned}$$

The second term can be bounded by using that $\partial _s \psi ^p = \ell ^{-2} p$ edgewise:

54 $$\begin{aligned} \ell ^{2} \Vert \partial _s \psi ^p \Vert _{L^2(\mathcal {E})}^2 = \ell ^{2} \Vert \ell ^{-2} p \Vert _{L^2(\mathcal {E})}^2= \Vert \ell ^{-1} p \Vert _{L^2(\mathcal {E})}^2. \end{aligned}$$

To handle the third term, involving jumps of the solution across vertices, we use the trace inequality: there exists $C_{t, i} > 0$ such that

55 $$\begin{aligned} \Vert \psi ^p_i \Vert _{L^2(\partial \Lambda _i)} \le C_{t,i} \Vert \partial _s \psi ^p_i \Vert _{L^2(\Lambda _i)} . \end{aligned}$$

The trace constant scales as $C_{t,i} \sim \ell _i^{-1/2}$. Thus

56 $$\begin{aligned} \ell ^{2} \Vert \alpha ^{-1} {[}\![\psi ^p]\!{]} \Vert _{L^2(\mathcal {V})}^2\le &  \ell ^{2} \sum _{\textbf{v}_j \in \mathcal {V}} \alpha _j^{-2} \sum _{\Lambda _i \in E(\textbf{v}_j)}\vert \psi ^p_i(\textbf{v}_j)\vert ^2 \nonumber \\\le &  \ell ^{2} \sum _{\textbf{v}_j \in \mathcal {V}} \alpha _j^{-2} \sum _{\Lambda _i \in E(\textbf{v}_j)} C_{t,i}^2 \Vert \partial _s \psi _i^p \Vert _{L^2(\Lambda _i)}^2 \nonumber \\\lesssim &  \ell ^{2}\Vert \partial _s \psi ^p \Vert _{L^2(\mathcal {E})}^2 \sum _{\textbf{v}_j \in \mathcal {V}} \alpha _j^{-2} \sum _{\Lambda _i \in E(\textbf{v}_j)} \ell _i^{-1} \nonumber \\= &  \Vert \ell ^{-1} p \Vert _{L^2(\mathcal {E})}^2 \sum _{\textbf{v}_j \in \mathcal {V}} \alpha _j^{-2} \sum _{\Lambda _i \in E(\textbf{v}_j)} \ell _i^{-1} \text { (using }(54)) \nonumber \\= &  \Vert \ell ^{-1} p \Vert _{L^2(\mathcal {E})}^2 \sum _{\textbf{v}_j \in \mathcal {V}} \frac{1}{m} \frac{\sum _{e_i \in E(\textbf{v}_j)} \ell _i}{\sum _{e_i \in E(\textbf{v}_j)} \ell _i} \nonumber \\= &  \Vert \ell ^{-1}p \Vert _{L^2(\mathcal {E})}^2. \end{aligned}$$

Combining (53)–(56) then gives (50), and thus we find that

57 $$\begin{aligned} \sup _{\psi \in V} \frac{b(\psi ,p)}{\Vert \psi \Vert _{V}}&\ge \frac{b(\psi ^p,p)}{\Vert \psi ^p \Vert _{V}} = \frac{(\nabla _\mathcal {G} \cdot \psi ^p,p)}{\Vert \psi ^p \Vert _{V}} =\frac{(\ell ^{-1} p, \ell ^{-1}p)_{L^2(\mathcal {G})}}{\Vert \psi ^p \Vert _{V}}\nonumber \\&=\frac{\Vert p \Vert _{M}^2}{\Vert \psi ^p \Vert _{V}} \ge \beta \Vert p \Vert _{M} \end{aligned}$$

and the inf-sup condition (49) holds. $\square$

#### Stability and robustness of families of dual discretizations

Next, we consider finite element discretizations $V^h \times M^h \subset V \times M$ of the dual mixed hydraulic network model given by (29) with (41). We let

58a $$\begin{aligned} V^h&= CG_k(\Lambda _1^h) \times CG_k(\Lambda _2^h) \times \dots \times CG_k(\Lambda _n^h), \end{aligned}$$

58b $$\begin{aligned} M^h&= DG_{k-1}(\Lambda ^h) \times \mathbb {R}^m , \end{aligned}$$

which correspond to branch-wise Raviart–Thomas-type spaces with the flux glued together using Lagrange multipliers at the internal vertices of the network. For the dual Stokes–Brinkman network model, we consider the same $V^h$ but instead consider continuous pressures; i.e., the pairing

59a $$\begin{aligned} V^h&= CG_k(\Lambda _1^h) \times CG_k(\Lambda _2^h) \times \dots \times CG_k(\Lambda _n^h), \end{aligned}$$

59b $$\begin{aligned} M^h&= CG_{k-1}(\Lambda ^h) \times \mathbb {R}^m, \end{aligned}$$

which correspond to branch-wise Taylor–Hood-type elements.

##### Remark 5

The dual mixed hydraulic network model constitutes the same variational formulation as was derived by Cerroni et al. (2018). Therein, the vertex values of *p* were introduced as Lagrange multipliers enforcing conservation of mass, and the system was discretized using branch-wise Taylor–Hood elements. However, our numerical experiments indicate that this choice is not inf-sup stable. Instead, we find that the hydraulic network model should be discretized using branch-wise Raviart–Thomas, while the dual mixed Stokes–Brinkman formulation could be discretized using branch-wise Taylor–Hood.

##### Remark 6

(Connection to non-conforming hybridized methods) We note that with (58) the discrete dual mixed formulation is closely related to the non-conforming (hybridized) mixed methods for the Darcy equation (Arnold and Brezzi 1985) where element-local $H(\text {div})$ spaces are glued across facets by Lagrange multipliers. Applying these ideas to our network setting, where the roles of elements/cells and facets are played respectively by graph edges and vertices, yields the norms (cf. 4.1)

60a $$\begin{aligned} \Vert q \Vert _{V^h}^2&= \Vert q \Vert _{L^2(\mathcal {E})}^2 + \Vert \partial _s q \Vert _{L^2(\mathcal {E})}^2 + \sum _{j=1}^m h^{-1}_j{[}\![q]\!{]} _j^2, \end{aligned}$$

60b $$\begin{aligned} \Vert p \Vert _{M^h}^2&= \Vert p_{\mathcal {E}} \Vert _{L^2(\mathcal {E})}^2 + \sum _{j=1}^m h_j p_{\mathcal {V}}^2. \end{aligned}$$

Here, for any internal vertex *b*, $h_j$ denotes the mean length of finite element cells in $\Lambda _i^h$ connected to *b*. Thus $h_j$ depends on the mesh and the degree of the node.

**Fig. 7:**
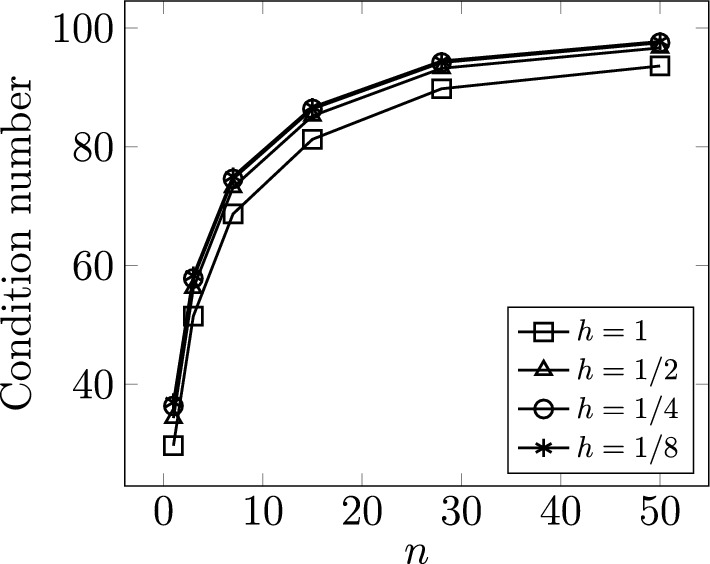
Condition numbers for the dual mixed discretizations with unweighted norms of $V\times M$, i.e. $l=1$, $\alpha =1$ in (4.1). Preconditioning based on these norms yields linear systems which become stiffer as the network length and complexity grow

**Fig. 8:**
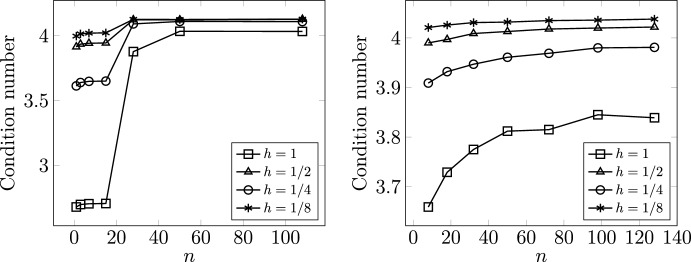
Condition numbers for the dual mixed discretizations with norms given by (4.1). Computations were performed on tree networks (left) and honeycomb networks (right), with *n* denoting the number of internal graph vertices and *h* denoting the mesh size. The resistance parameter was set to $\mathcal {R}=1$

Now, we examine numerically the robustness and conditioning of the dual discretization. Using lowest order elements $k=1$ in the family of discretizations (58), Figs. 7 and 8 report respectively the condition numbers of the dual mixed formulation using the unweighted norms (in particular the *V* norm (24)) and the domain-dependent norms (4.1). In both cases the condition numbers appear to be stable in *h*, however, only the weighted norms lead to boundedness also in the number of bifurcations for different graph configurations (tree, honeycomb). Note that the length of the graph $\ell$ increases with the number of generations in these graph configurations. For the honeycomb networks, $\ell$ grows from approximately $\ell =6$ to $\ell =16$ between the first and final generations, while for the tree graphs $17< \ell < 50$.

Let us finally comment on robustness and stability with respect to the resistance parameter. This is of particular interest for simulations of flow in branching networks, where the cross-section size typically reduces at each branching generation. In this case, we may apply results from $\mathcal {R}$-robust Darcy preconditioners (Badia and Codina 2010) to propose the following norms for the solution spaces (instead of (4.1)):

61a $$\begin{aligned} \Vert q \Vert ^2_{V}&= \Vert \mathcal {R}^{1/2} q \Vert ^2_{L^2(\mathcal {E})} + \Vert \ell \mathcal {R}^{1/2} \partial _s q \Vert ^2_{L^2(\mathcal {E})} + \Vert \ell \mathcal {R}_j^{1/2} \alpha ^{-1}{[}\![q]\!{]} \Vert _{L^2(\mathcal {V})}^2 , \end{aligned}$$

61b $$\begin{aligned} \Vert p \Vert ^2_{M}&= \Vert \ell ^{-1} \mathcal {R}^{-1/2} p_{\mathcal {E}} \Vert ^2_{L^2(\mathcal {E})} + \Vert \ell ^{-1} \alpha \mathcal {R}_j^{-1/2} p_{\mathcal {V}} \Vert _{L^2(\mathcal {V})}^2. \end{aligned}$$

**Fig. 9:**
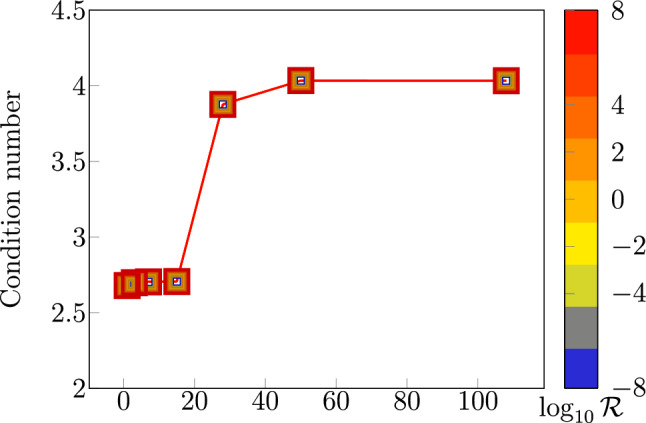
Conditioning of the dual mixed formulation with norm (61) for different values of spatially constant resistance parameter (encoded by color). Tree networks are considered with *n* denoting the number of internal graph vertices. The mesh size is fixed at $h=1$. The parameter dependent norm (61) results in condition numbers practically independent of $\mathcal {R}$ as the corresponding curves overlap

Here $\mathcal {R}_j$ represents the mean resistance at bifurcation point $\textbf{v}_j$ defined by averaging over connected branches. The robustness of the mixed formulation with norms (61) is demonstrated numerically in Fig. 9.

### Approximation and convergence of primal and dual discretizations

To examine the approximation properties of the primal and dual mixed hydraulic network models, we compute the error and convergence rates against an analytic solution of a simple bifurcation problem. To be more precise, let $\textbf{v}_1=(0,0)$, $\textbf{v}_1=(0,0.5)$, $\textbf{v}_2=(-0.5,1)$ and $\textbf{v}_3=(0.5,1)$. From these vertices, we create a Y-shaped (bifurcating) graph by setting $e_1=(\textbf{v}_1, \textbf{v}_2)$, $e_2=(\textbf{v}_2, \textbf{v}_3)$ and $e_2=(\textbf{v}_2, \textbf{v}_3)$. Each edge is associated with a resistance $\mathcal {R}=1$ and a cross-section area $A=1$. Letting *s* denote the distance from the root node $\textbf{v}_1$, we take

$$\begin{aligned} q&= {\left\{ \begin{array}{ll} 1 + \cos (\pi s) +\sin (2\pi s) & \text { on } e_1,\\ \frac{1}{2} + \cos (\pi s) +\sin (2\pi s) & \text { on } e_2, e_3, \end{array}\right. } \\ p&= \sin (\pi s)+\cos (2\pi s) \text { on } e_1,e_2,e_3 . \end{aligned}$$

as analytic solutions; inserting these in (27) gives the associated values for *f* and *g*. Finally, we use the analytic solution pressure to impose suitable pressure boundary conditions. We note that *p* is smooth on all of $\Lambda$. Contrarily, *q* is smooth on all edges $\Lambda _i$, but discontinuous across the bifurcation.

Table 5 shows the errors and convergence rates associated with these discrete solutions. The primal mixed approximation shows order *k* convergence for $k = 1, 2, 3$ for both flux and pressure, measured in the $L^2(\Lambda )$- and $H^1(\Lambda )$-norms, respectively. This agrees with the expected rates for standard finite element methods (Brenner and Scott 2002).

For $k=1$, the dual mixed approximation similarly shows order one convergence of the pressure and flux, now measured in the $L^2$- and $H(\textrm{div}; \mathcal {G})$-norms, respectively. Increasing the degree, we find that the flux approximation enjoys *k*-order convergence in the $H(\textrm{div}; \mathcal {G})$-norms, while the pressure error converges at a maximum rate of two. Egger and Philippi (2023)[Lemma 4] showed that higher order convergence for a similar numerical method is possible in a single vessel. Indeed, repeating the convergence test on a single vessel (no bifurcations), we found that the optimal *k*-order convergence was restored. The lack of higher-order convergence is therefore likely a consequence of the bifurcation condition.

**Table 5:**
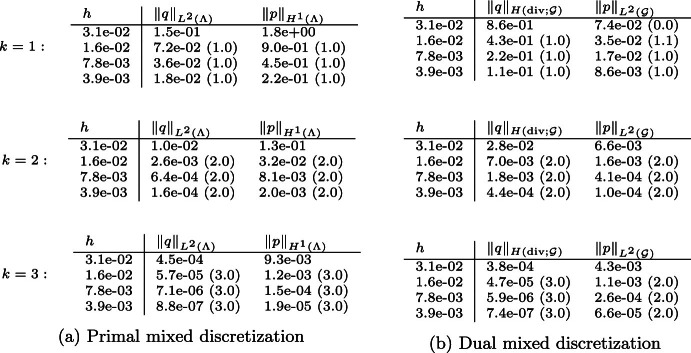
Approximation errors (and convergence rates) of the (left) primal mixed discretization (32) and (right) dual mixed hydraulic network discretization (41) for an idealized test case, with discrete spaces given by (39) and (58), respectively, and for $k = 1, 2, 3$

We observe optimal convergence orders for the approximation of the velocity *q* for both formulations and all *k*, and for the pressure *p* for both formulations for $k = 1, 2$. For $k = 3$, the primal formulation yields optimal rates also for *p*, while the dual formulation is one order suboptimal

## Discussion

The results of this paper are three-fold. First, we present a rigorously derived Stokes–Brinkman network model for representing fluid flow in open or porous PVSs with generalized annular cross-sections. Second, we study the existence, uniqueness and stability of solutions and numerical approximations to these equations. Specifically, we prove that the approximations converge uniformly with respect to the network topology and cardinality in appropriately weighted norms. Third, by simulating CSF flow in perivascular networks, we find that uniform wave pulsations may induce directional net flow given sufficient fluid influx and efflux pathways.

In terms of modelling limitations, we here consider only motion of the inner perivascular wall, ignoring the elasticity of the surrounding tissue. Moreover, all simulations assume the cross-section to be an annular circle, and we do not model pressure interactions between the PVS network flux and the surrounding tissue. Both of these aspects would be expected to reduce the net flow observed within the network. In the simulations of the arterial-capillary-venous network, all vessels including capillaries and veins pulsate, which can also be considered an extreme case.

The model and numerical methods presented here provide a robust and computationally efficient approach to simulate perivascular flow in non-trivial networks. The simulation code, built on (Gjerde 2022), is openly available (Gjerde and Kuchta 2023) and provides a solid technological foundation for further computational studies of perivascular fluid flow and transport.

## Derivation of the reduced model

In this section, we will show how the three-dimensional Stokes Eq. (3b) can be reduced to the one-dimensional Stokes–Brinkman Eq. (10a)–(10b). Let $\bar{f}=\bar{f}(s)$ denote the average of a function *f* over the cross-section boundary $\partial C$,

62 $$\begin{aligned} \bar{f}= \frac{1}{\vert \partial C \vert } \int _{\partial C} f \, \textrm{d} \theta , \end{aligned}$$

and let $\bar{\bar{f}}=\bar{\bar{f}}(s)$ denote the average over the cross-section *C*

63 $$\begin{aligned} \bar{\bar{f}} = \frac{1}{A} \int _{C} f r\, \textrm{d} r\, \textrm{d} \theta . \end{aligned}$$

Recalling that the reduced model is posed in terms of the cross-section pressure and the cross-section flux, we can now write this as

64 $$\begin{aligned} p = \bar{\bar{p}}^{\text {ref}} \text { and } q=A \bar{\bar{v}}_s^{\text {ref}}. \end{aligned}$$

Let *R* be a function so that $R = R^1$ on the inner boundary and $R=R^2$ on the outer boundary of *C*. To move derivatives out of the integrals, we will make use of Reynolds transport theorem, which yields the following relations:

65 $$\begin{aligned} \int _{C} \partial _{s} f \, r \, \textrm{d} r\, \textrm{d} \theta&= \partial _{s} \bar{\bar{f}} - \int _{\partial C} \partial _{s} R f \, \textrm{d} \theta , \end{aligned}$$

66 $$\begin{aligned} \int _{C} \partial _{t} f \, r \, \textrm{d} r\, \textrm{d} \theta&= \partial _{t} \bar{\bar{f}} - \int _{\partial C} w f(s,R,\theta ) \, \textrm{d} \theta , \end{aligned}$$

where $$w=\textbf{w}\cdot \textbf{n}$$.

### Reduced conservation equation

Consider a single channel with centerline $\Lambda$. In this section, we show how the conservation equation $\nabla \cdot \textbf{v}^{\text {ref}}=0$ can be reduced to a one-dimensional equation for the cross-section flow.

Recall the Frenet-Serret frame of $\Lambda$ with $\textbf{T}, \textbf{B}, \textbf{N}$. We denote by *X*(*s*), *Y*(*s*) the cylindrical coordinate system associated with the normal and binormal vectors $\textbf{B}$ and $\textbf{N}$. We decompose $\textbf{v}^{\text {ref}}=(v_r^\text {ref}, v_\theta ^{\text {ref}}, v_s^{\text {ref}})$, where $v_r^\text {ref}$ and $v_\theta ^{\text {ref}}$ denote the radial and angular components of $\textbf{v}^{\text {ref}}$ with respect to *X*, *Y*, and $v_s^{\text {ref}}$ is the component of $\textbf{v}^{\text {ref}}$ in the tangent direction $\textbf{T}$.

In the cylindrical coordinate system, the conservation equation then reads:

$$\begin{aligned} \partial _s v_s^{\text {ref}} + \frac{1}{r}\partial _{\theta } v_\theta ^{\text {ref}} + \frac{1}{r} \left( \partial _{r} \left( r v_r^\text {ref} \right) \right) =0. \end{aligned}$$

Fixing $s \in (0, l)$, and integrating over the cross-section *C*(*s*), we find

$$\begin{aligned} \int _{C} \partial _s v_s^{\text {ref}} r\, \textrm{d} r\, \textrm{d} \theta&= \partial _s \int _{C} v_s^{\text {ref}} r\, \textrm{d} r\, \textrm{d} \theta - \int _{\partial C} \partial _s R \underbrace{v_s^{\text {ref}}}_{=0} \, r \, \textrm{d} \theta = \partial _s q, \\ \int _{C} \frac{1}{r}\partial _{\theta } v_\theta ^{\text {ref}} r\, \textrm{d} r\, \textrm{d} \theta&= \int _{R^1}^{R^2} \int _0^{2\pi } \partial _{\theta } v_\theta ^{\text {ref}} \, \textrm{d} \theta \, \textrm{d} r=\int _{R^1}^{R^2} \underbrace{v_\theta ^{\text {ref}}\vert _{\theta =2\pi }-v_\theta ^{\text {ref}}\vert _{\theta =0}}_{= 0} \, \textrm{d} r= 0, \\ \int _{C} \frac{1}{r} \left( \partial _{r} \left( r v_r^\text {ref} \right) \right) r\, \textrm{d} r&= \int _{0}^{2\pi } \int _{R^1}^{R_2} \partial _{r} \left( r v_r^\text {ref} \right) \, \textrm{d} r\, \textrm{d} \theta = \int _{\partial C } R v_r^\text {ref} \, \textrm{d} \theta \\&=\int _{\partial C } R w \, \textrm{d} \theta = \vert \partial C \vert \, \bar{w}. \end{aligned}$$

For the first term, we used (65) and that $v_s^{\text {ref}}=0$ on $\partial C$ (due to the no-slip boundary condition on $\Gamma$). For the second term, we used the fundamental theorem of calculus. For the third term, we used $v_r^\text {ref}=w$ on $\Gamma$.

Inserting this in the conservation of mass equation yields

67 $$\begin{aligned} \begin{aligned} \int _{C} \left( \partial _s v_s^{\text {ref}} + \frac{1}{r} \left( \partial _{r} \left( r v_r^\text {ref} \right) \right) \right) r\, \textrm{d} r&= \partial _s q- \vert \partial C \vert \bar{w} = 0. \end{aligned} \end{aligned}$$

Using that $\partial _t A = \vert \partial C \vert \bar{w}$, the integrated conservation Eq. (67) then reads

68 $$\begin{aligned} \partial _s q+ \partial _t A=0. \end{aligned}$$

This equation is well known in the context of blood flow models (Olufsen 1999; Formaggia et al. 2003), and simply states that changing the size of the cross-section will drive a cross-section flux. One may notice that this an exact result, meaning that the reduction holds without any assumptions on $p^{\text {ref}}$ and $v^{\text {ref}}$.

### Reduced axial momentum equation

Recall the assumption (8b), stating that $v_s^\text {ref}=\hat{v}(s,t) v^{vp} (r,\theta ;s,t)$, where $v^{vp}(r,\theta ;s,t)$ is the velocity profile associated with the cross-section $C=C(s,t)$. In this section, we show how this assumption can be used to derive a reduced momentum equation.

The (full) axial momentum equation reads $v_s^{\text {ref}}$ reads

$$\begin{aligned} \partial _t v_s^{\text {ref}} - \frac{\nu }{\varphi } \Delta v_s^{\text {ref}} + \frac{\nu }{\kappa } v_s^{\text {ref}} + \partial _s p&= 0. \end{aligned}$$

The dimension reduction is performed by integrating this equation over an arbitrary portion $\tilde{\Omega }$ of the annular cylinder,

$$\begin{aligned} \int _{\tilde{\Omega }}\left( \partial _t v_s^{\text {ref}} - \frac{\nu }{\varphi } \Delta v_s^{\text {ref}} + \frac{\nu }{\kappa } v_s^{\text {ref}} + \partial _s p \right) \, r \, \textrm{d} r\, \textrm{d} \theta \, \textrm{d} s&= 0, \end{aligned}$$

and using Reynolds transport theorem to transfer the derivatives out of the integral.

We evaluate the results of (69) term by term. For the first term,

$$\begin{aligned} \int _{\tilde{\Omega }} \partial _t v_s^{\text {ref}} \, r \, \textrm{d} r\, \textrm{d} \theta \, \textrm{d} s&= \int _{s_1}^{s_2} \int _{C} \partial _t v_s^{\text {ref}} r\, \, \textrm{d} r\, \textrm{d} \theta \, \textrm{d} s\\&= \int _{s_1}^{s_2} \left( \partial _t \int _{C} v_s^{\text {ref}} r\, \, \textrm{d} r\, \textrm{d} \theta - \int _{\partial C} \underbrace{v_s^{\text {ref}}}_{=0 \text { on} \Gamma } w R d\theta \right) \, \textrm{d} s= \int _{s_1}^{s_2} \partial _t q \, \textrm{d} s, \end{aligned}$$

where we used (66) to move the time derivative out of the integral.

For the second term, we apply the divergence theorem, which yields

$$\begin{aligned} \int _{\tilde{\Omega }} \Delta v_s^{\text {ref}} \, r\, \textrm{d} r\, \textrm{d} \theta \, \textrm{d} s&= \int _{\partial \tilde{\Omega }} \nabla v_s^{\text {ref}} \cdot \textbf{n}\, \textrm{d}\varvec{\sigma } \\&= \underbrace{\int _{\textrm{C}(s_2,t)} \partial _s v_s^{\text {ref}} r\, \textrm{d} r\, \textrm{d} \theta - \int _{\textrm{C}(s_1,t)} \partial _s v_s^{\text {ref}} r\, \textrm{d} r\, \textrm{d} \theta }_{\text {top and bottom boundary}} \\&\quad +\underbrace{\int _{s_1}^{s_2} \int _{\partial C(s,t)} \nabla v_s^{\text {ref}} \cdot \textbf{n}\, \textrm{d} \theta \, \textrm{d} s}_{\text {inner and outer lateral boundary}}, \end{aligned}$$

where $\textrm{C}(s_2,t)$ and $\textrm{C}(s_1,t)$ denote the top and bottom boundaries of $\tilde{\Omega }$. For the top and bottom boundary terms, we compute

$$\begin{aligned}&\int _{C(s_2,t)} \partial _s v_s^{\text {ref}} \,r\, \textrm{d} r\, \textrm{d} \theta - \int _{C(s_1,t)} \partial _s v_s^{\text {ref}} \,r\, \textrm{d} r\, \textrm{d} \theta \\&\quad = \int _0^{2\pi } \left( \int _{R^{1}(s_2,\theta ,t)}^{R^{2}(s_2,\theta ,t)} \partial _s v_s^{\text {ref}}(s_2,t) r \, \, \textrm{d} r- \int _{R^{1}(s_1,\theta ,t)}^{R^{2}(s_1,\theta ,t)} \partial _s v_s^{\text {ref}}(s_1,t) r \, \, \textrm{d} r\right) \, \textrm{d} \theta \\&\quad =\partial _s \int _0^{2\pi } \left( \int _{R^{1}(s_2,\theta ,t)}^{R^{2}(s_2,\theta ,t)} v_s^{\text {ref}}(s_2,t) r \, \, \textrm{d} r- \int _{R^{1}(s_1,\theta ,t)}^{R^{2}(s_1,\theta ,t)} v_s^{\text {ref}}(s_1,t) r \, \, \textrm{d} r\right) \, \textrm{d} \theta \\&\quad =\partial _s \left( q(s_2,t) -q(s_1,t) \right) \\&\quad =\partial _s \int _{s_1}^{s_2} \partial _s( q(s,t))\, \textrm{d} s\\&\quad = \int _{s_1}^{s_2} \frac{\partial ^2}{\partial s^2} q(s,t)\, \textrm{d} s\end{aligned}$$

where we moved the partial derivative $\partial _s$ directly out of the integral as the integration domains $C(s_1)$ and $C(s_2)$ do not depend on *s*.

For the lateral boundary terms, we recall the splitting (8b), which implies $\nabla v_s^{\text {ref}} = (\partial _r, \partial _\theta , \partial _s) v_s^{\text {ref}} = (\hat{v}\partial _r v^{vp}, \hat{v}\partial _\theta v^{vp}, v^{vp}\partial _s \hat{v})$. On the inner and outer lateral boundaries, the outward pointing unit normal is perpendicular to the axial direction. Thus $\nabla v_s^{\text {ref}} \cdot \textbf{n}= \nabla v^{vp} \cdot \textbf{n}$. From this we find

$$\begin{aligned} \int _{s_1}^{s_2} \int _{\partial C} \nabla v_s^{\text {ref}} \cdot \textbf{n}\,\textrm{d}\sigma \, \textrm{d} s&= \int _{s_1}^{s_2} \hat{v}\int _{\partial C} \nabla v^{vp} \cdot \textbf{n}\,r\, \textrm{d} r\, \textrm{d} \theta \, \textrm{d} s\\&= \int _{s_1}^{s_2} \hat{v}\int _{C} \Delta v^{vp} \,r\, \textrm{d} r\, \textrm{d} \theta \, \textrm{d} s\\&= \int _{s_1}^{s_2} \hat{v} A \overline{\overline{\Delta v^{vp}} } \, \textrm{d} s\end{aligned}$$

where we used the divergence theorem again.

By a straightforward calculation, we have

$$\begin{aligned} q&= \int _0^{2\pi } \int _{R^{1}}^{R^{2}} v_s^{\text {ref}} \, r \, \textrm{d} r\, \textrm{d} \theta = \hat{v}\int _0^{2\pi } \int _{R^{1}}^{R^{2}} v^{vp} \, r \, \textrm{d} r\, \textrm{d} \theta = \hat{v} A \bar{\bar{v}}^{vp} \\ \Rightarrow \hat{v}&= \frac{q}{A \bar{\bar{v}}^{vp}}. \end{aligned}$$

Consequently

$$\begin{aligned} \int _{\tilde{\Omega }} \frac{\nu }{\varphi } \Delta v_s^{\text {ref}} \, r\, \textrm{d} r\, \textrm{d} \theta \, \textrm{d} s&= \int _{s_1}^{s_2} \frac{\nu }{\varphi } \frac{\partial ^2}{\partial s^2} q- \frac{\nu }{\varphi } \frac{\overline{\overline{\Delta v^{vp}}} }{\bar{\bar{v}}^{vp}} q\, \textrm{d} s. \end{aligned}$$

For the third term (the Brinkman term), we have by definition

$$\begin{aligned} \int _{\tilde{\Omega }} \frac{\nu }{\varphi } v_s^{\text {ref}} \, r\, \textrm{d} r\, \textrm{d} \theta \, \textrm{d} s= \int _{s_1}^{s_2} \frac{\nu }{\kappa } q\, \textrm{d} s. \end{aligned}$$

For the fourth and final term (the pressure term), we assumed $p=p(s,t)$; thus, we simply have

$$\begin{aligned} \int _{\tilde{\Omega }} \partial _s p \, r\, \textrm{d} r\, \textrm{d} \theta \, \textrm{d} s&= \int _{s_1}^{s_2} \partial _s p \int _0^{2\pi } \int _{R^{1}}^{R^{2}} 1 \, r\, dr \, \textrm{d} \theta \, \textrm{d} s= \int _{s_1}^{s_2} \partial _s p \, \textrm{d} s. \end{aligned}$$

This yields the following integrated momentum equation:

$$\begin{aligned} \int _{s_1}^{s_2} \partial _t q- \frac{\nu }{\varphi } \partial _{ss} q- \frac{\nu }{\varphi } \frac{\overline{\overline{\Delta v^{vp}} }}{\bar{\bar{v}}^{vp}} q+ \frac{\nu }{\kappa } q+ \partial _s p \, \textrm{d} s=0. \end{aligned}$$

As this holds for arbitrary $s_1, s_2$, we have the following averaged momentum equation for $v_s^{\text {ref}}$:

69 $$\begin{aligned} \partial _{t} q- \frac{\nu }{\varphi } \partial _{ss} q+ \frac{\nu }{\varphi } \frac{\overline{\overline{\Delta v^{vp}} }}{\bar{\bar{v}}^{vp}} q+\frac{\nu }{\kappa } q+ \partial _s p =0. \end{aligned}$$

with

70 $$\begin{aligned} \mathcal {R}(s,t) = \frac{\nu }{\varphi } \frac{\overline{\overline{\Delta v^{vp}}}}{\bar{\bar{v}}^{vp}} + \frac{\nu }{\kappa }. \end{aligned}$$

In the next section, we show how the velocity profile $v^{vp}$ (and hence the resistance $\mathcal {R}$) can be computed for a cross-section *C*.

#### Computing the resistance

Consider a domain with a single, straight unit length centerline aligned with the *z*-axis and a constant cross-section *C*:

$$\begin{aligned} \Omega = \{ (r\cos (\theta ),r\sin (\theta ),s): R^1(\theta )<r<R^2(\theta ), 0<s<l \}. \end{aligned}$$

Next, we assume the flow in this domain is independent of time and driven by some constant pressure drop. Then $p=p(s)$, and we have $\nabla p=(0,0,\partial _s p)=(0,0,-c)$. Inserting this in (3b), we see that the flow $\textbf{v}$ is purely axial, i.e. $\textbf{v}= (0,0,v_s^{\text {ref}})$. Moreover, from conservation of mass, we have

$$\begin{aligned} \nabla \cdot \textbf{v}= (0,0, \partial _s v_s^{\text {ref}}) = 0 \quad \Rightarrow \quad v_s^{\text {ref}}=v_s^{\text {ref}}(r,\theta ). \end{aligned}$$

In this case, we have the splitting $v^{\text {ref}}=\hat{v}v^{vp}(r,\theta )$, where $\hat{v}$ is a constant.

Inserting this in the axial component of the momentum equation in the Stokes–Brinkman system (3b), we find

71 $$\begin{aligned} - \frac{\nu }{\varphi } \Delta v_s^{\text {ref}} + \frac{\nu }{\kappa }v_s^{\text {ref}} = \frac{c}{\hat{v}}. \end{aligned}$$

Notice that the scaling $\hat{v}$ in (8b) is arbitrary; we now fix it so that $c/(\nu \hat{v})=1$. The velocity profile associated with a cross-section *C* can then be obtained by solving

72 $$\begin{aligned} \begin{aligned} - \Delta v^{vp} + \frac{1}{\kappa } v^{vp}&= -1 &  \text { in } C, \\ v^{vp}&= 0 &  \text { on } \partial C. \end{aligned} \end{aligned}$$

Averaging the first equation in (72) yields

$$\begin{aligned} \overline{\overline{\Delta v^{vp}}}&= 1+\frac{1}{\kappa } \overline{\overline{v^{vp}}}. \end{aligned}$$

Inserting this in (70), we find

73 $$\begin{aligned} \mathcal {R}(s,t) = \frac{\nu }{q^{vp}} + 2 \frac{\nu }{\kappa }, \end{aligned}$$

where $q^{vp}=\bar{\bar{v}}^{vp}$ is the velocity profile cross-section flux.

##### Remark 7

For open channels ($\kappa \rightarrow \infty$), this result agrees with the one derived in (Tithof et al. 2019). Specifically, they non-dimensionalize the Stokes equations to derive the resistance

74 $$\begin{aligned} \mathcal {R} = \frac{\nu }{q^{vp}} \frac{1}{(R^1)^4 }. \end{aligned}$$

#### Reduced boundary and bifurcation conditions

To derive the reduced boundary conditions, we simply average the traction boundary condition (5) over the cross-section. This yields

75 $$\begin{aligned} \frac{1}{A} \int _{C} ( \frac{\nu }{\varphi } \partial _s v_s^{\text {ref}}-p^{\text {ref}})\, r \, \textrm{d} r= \frac{1}{A} \left( \frac{\nu }{\phi } q- p\right) = \tilde{p}^{\text {ref}}. \end{aligned}$$

## Footnotes

1 For the image-based cross-sections in Fig. 2, it is not trivial to understand how the cross-section deforms as the inner radius shrinks or expands. Consequently, it is non-trivial to acquire reliable estimates of the resulting cross-sectional area and resistance. The directional flow is highly sensitive to variations in these two parameters. For this reason, all simulations of directional PVS flow have been performed assuming a circular, annular cross-section, for which the cross-section area and resistance are readily available.
